# Modeling the impact of obesity on the lifetime risk of chronic kidney disease in the United States using updated estimates of GFR progression from the CRIC study

**DOI:** 10.1371/journal.pone.0205530

**Published:** 2018-10-19

**Authors:** Benjamin O. Yarnoff, Thomas J. Hoerger, Sundar S. Shrestha, Siobhan K. Simpson, Nilka R. Burrows, Amanda H. Anderson, Dawei Xie, Hsiang-Yu Chen, Meda E. Pavkov, the CRIC Study Investigators

**Affiliations:** 1 RTI International, Research Triangle Park, North Carolina, United States of America; 2 Centers for Disease Control and Prevention, Atlanta, Georgia, United States of America; 3 Tulane University, New Orleans, Louisiana, United States of America; 4 University of Pennsylvania, Philadelphia, Pennsylvania, United States of America; International University of Health and Welfare, School of Medicine, JAPAN

## Abstract

**Rationale & objective:**

As the prevalence of obesity continues to rise in the United States, it is important to understand its impact on the lifetime risk of chronic kidney disease (CKD).

**Study design:**

The CKD Health Policy Model was used to simulate the lifetime risk of CKD for those with and without obesity at baseline. Model structure was updated for glomerular filtration rate (GFR) decline to incorporate new longitudinal data from the Chronic Renal Insufficiency Cohort (CRIC) study.

**Setting and population:**

The updated model was populated with a nationally representative cohort from National Health and Nutrition Examination Survey (NHANES).

**Outcomes:**

Lifetime risk of CKD, highest stage and any stage.

**Model, perspective, & timeframe:**

Simulation model following up individuals from current age through death or age 90 years.

**Results:**

Lifetime risk of any CKD stage was 32.5% (95% CI 28.6%–36.3%) for persons with normal weight, 37.6% (95% CI 33.5%–41.7%) for persons who were overweight, and 41.0% (95% CI 36.7%–45.3%) for persons with obesity at baseline. The difference between persons with normal weight and persons with obesity at baseline was statistically significant (p<0.01). Lifetime risk of CKD stages 4 and 5 was higher for persons with obesity at baseline (Stage 4: 2.1%, 95% CI 0.9%–3.3%; stage 5: 0.6%, 95% CI 0.0%–1.1%), but the differences were not statistically significant (stage 4: p = 0.08; stage 5: p = 0.23).

**Limitations:**

Due to limited data, our simulation model estimates are based on assumptions about the causal pathways from obesity to CKD, diabetes, and hypertension.

**Conclusions:**

The results of this study indicate that obesity may have a large impact on the lifetime risk of CKD. This is important information for policymakers seeking to set priorities and targets for CKD prevention and treatment.

## Introduction

The prevalence of obesity (body mass index [BMI] ≥ 30 kg/m^2^) in the United States has increased significantly since the 1980s, contributing to higher risk for chronic diseases and mortality.[1–3] As a major risk factor for diabetes, hypertension, and cardiovascular disease, obesity is an important contributor to the risk for chronic kidney disease (CKD).[4–8] Furthermore, the risk of CKD onset and progression may be directly increased by obesity through adaptive glomerular changes that frequently evolve into pathologic alterations.[9] These observations suggest that obesity may have a significant impact on the lifetime risk of CKD. However, the evidence for an independent association (i.e. apart from the impact of obesity on hypertension and diabetes) between obesity and CKD is mixed.[8, 10–13] Further, data must contain observations of persons over a long time period to assess the impact of risk factors such as obesity on the lifetime risk of CKD.

The long-term dynamics of these pathways on the lifetime risk of CKD is best assessed with a simulation model, because the length of time required to estimate lifetime risk with observed data is not feasible. The CKD Health Policy Model, a microsimulation model of CKD progression, has been used previously to examine the burden of CKD, and has been extensively validated.[14] Here we used this model to examine the impact of obesity on the lifetime risk of CKD.

## Methods

This study used the CKD Health Policy Model, a microsimulation model of CKD progression.[14–17] Briefly, the model simulates progression of CKD and its complications in a nationally representative cohort drawn from the National Health and Nutrition Examination Survey (NHANES) through age 90 years or death. The model includes eight states: no CKD, CKD stages 1 through 5 (with stage 3 divided into 3a and 3b), and death. CKD stages are defined by estimated glomerular filtration rates (eGFR) and kidney damage as measured by the presence of albuminuria (urinary albumin to creatinine ratio ≥30 mg/g).[18] CKD stages are largely sequential based on GFR, but stages 1 and 2 also require kidney damage. So, in the simulations, persons who reach stage 3b must first go through stage 3a; similarly, those who reach stage 4 must go through stages 3a and 3b, and those who reach stage 5 must go through stages 3a, 3b, and 4. However, it is possible to reach stage 3a or higher without going through stages 1 and 2 if a person never has kidney damage as measured by elevated albuminuria. The model concomitantly simulates the natural history of complications from CKD. Model parameters are derived from the epidemiological literature, clinical trials, and new analysis of eGFR progression from the Chronic Renal Insufficiency Cohort (CRIC) study.

### Change in eGFR

Previously, the eGFR component of the model was based on another cost-effectiveness study by Boulware et al.[19] This structure used the mean change in eGFR over time for persons with different sets of risk factors (diabetes, hypertension, and proteinuria). For this study, we updated this component of the model, because model parameters were potentially out of date and the structure was deterministic with no inclusion of the variation in eGFR change across persons with the same risk factors. Variation in eGFR change is potentially an important driver of outcomes, because progression to severe stages of CKD such as stage 5 and ESRD is likely driven by the tail of the distributions rather than the mean.

To update the eGFR component of the model, we analyzed data on eGFR change from the CRIC study. The CRIC study is a longitudinal study of persons with CKD that began in 2001 with regular follow-up over 7 to 14 years. It was established to help inform the understanding of CKD and related cardiovascular disease. Importantly, one of its primary goals is to examine risk factors for decline in eGFR. The analysis sample from the CRIC study consisted of 3,302 persons. Table 1 presents descriptive statistics for the sample.

**Table 1:** Baseline characteristics of the Chronic Renal Insufficiency Cohort study Analytic sample.

*Variable*	*N (%)*
Sex	
Male	1803 (54.60)
Female	1499 (45.40)
Race-ethnicity	
Non-Hispanic white	1463 (44.31)
Non-Hispanic black	1364 (41.31)
Hispanic	347 (10.51)
Other	128 (3.88%)
Age (years)	
30 to 49	617 (18.69)
50 to 64	1714 (51.91)
≥65	971 (29.41%)
Urine protein (g/24 hours)	
<0.10	1277 (38.67)
0.10–0.49	977 (29.59)
0.50–1.49	499 (15.11)
≥1.50	549 (16.63)
Diabetes	1549 (46.91)
Hypertension	2830 (85.71)
BMI (kg/m^2^)	
<18.5 (underweight)	18 (0.55)
18.5–24.9 (normal)	481 (14.57)
25–29.9 (overweight)	951 (28.80)
≥30 (obesity)	1852 (56.09)
Baseline eGFR <60 ml/min/1.73m^2^	2794 (84.62)

Abbreviations: BMI = Body Mass Index; eGFR = estimated glomerular filtration rate

We used a mixed-effect regression model to estimate the association of clinical risk factors (hypertension, diabetes, proteinuria, and obesity) and demographic factors (sex and race) with annual change in eGFR and the variance of annual change in eGFR. We estimated the regression equations separately for persons with eGFR < 60 at baseline and eGFR ≥ 60 at baseline. We took coefficients from these regression equations as model parameters for the annual change in eGFR associated with sex, race, hypertension, diabetes, proteinuria, and obesity. We took the estimated variance of the error term in the mixed-effect model as the standard deviation of the distribution of annual change in eGFR. Table 2 lists model parameters for annual change in eGFR. After updating model parameters, we performed model calibration by comparing simulated output to CKD prevalence estimates in NHANES and comparing simulated and actual eGFR decline for a cohort from the Atherosclerosis Risk in Communities (ARIC) study. We found that simulated estimates of the ARIC cohort lined up well with actual eGFR decline for persons with baseline eGFR < 60, but overestimated the decline for persons with baseline eGFR ≥ 60. This is a logical outcome, because the sample of persons in the CRIC study with baseline eGFR ≥ 60 was small and not designed to be representative of that group in the broader population. Therefore, estimates of the variance in eGFR decline for that group in the sample was much larger than observed in the ARIC cohort. We calibrated the model to reflect this with a multiplier to bring the variance term for persons with baseline eGFR ≥ 60 in line with the variance observed for this group in the ARIC cohort.

**Table 2:** Model parameters for annual change in eGFR based on person characteristics and conditions and baseline eGFR.

Person Characteristics and Conditions	Annual Change in eGFR for Persons with the Characteristic of Condition (ml/min/1.73m^2^)	
	If Baseline eGFR < 60	If Baseline eGFR ≥ 60
Base annual change in eGFR	0.42	0.33
Annual change in eGFR for Demographic Characteristics and Risk Factors		
Sex: Female	-0.12	0.14
Race: Non-Hispanic black	-0.61	-0.17
Race: Hispanic	-0.40	0.37
Race: Other	-0.65	-0.28
Proteinuria: 0.10—<0.50 g/24 hours	-0.86	-0.26
Proteinuria: 0.50—<1.50 g/24 hours	-2.38	-1.05
Proteinuria: ≥1.50 g/24 hours	-4.17	-4.56
Diabetes	-0.29	-0.81
Hypertension	-0.15	-0.29
Obesity (BMI ≥ 30 kg/m^2^)	0.26	-0.15
Standard Deviation of distribution of annual change in eGFR	1.64	1.77

Abbreviations: BMI = Body Mass Index; eGFR = estimated glomerular filtration rate. Notes: The parameter values are based on the coefficients from a mixed-effect model of slope of eGFR in the Chronic Renal Insufficiency Cohort Study population. Parameters represent the annual change in eGFR for persons with each characteristic and condition. All characteristics and conditions in the table were included together in the mixed-effect estimation.

### Overweight and obesity

We updated the model by adding an obesity module that modeled the impact of overweight and obesity on diabetes, hypertension, and cardiovascular disease (CVD) and annual changes in BMI (Table 3). Parameters for the impact on risk factors and CVD are drawn from Wilson et al.[4] which used data from the Framingham study to estimate the relationship between obesity and other conditions. We considered including parameters for the effect of obesity on non-CVD mortality, but found that studies with a full set of controls estimated no effect on non-CVD mortality.[4] Finally, we updated the model to simulate changes in BMI over time. We conducted a literature review to identify studies that estimated average annual change in BMI. From this literature review we identified parameters for average annual change in BMI based on age, sex, and race (Table 3). We calibrated the model based on simulated versus actual change in BMI for the ARIC cohort.

**Table 3:** Model parameters for relative risks related to obesity and annual change in BMI.

Relative Risk and Annual Change Model Parameters	Parameter Values	Source of Parameter Values
Relative Risk for Diabetes from		Wilson et al. 2002[4]
Overweight (BMI 25 kg/m^2^ to 29.9 kg/m^2^)	Men: 1; Women: 1	
Obesity (BMI 30+ kg/m^2^)	Men: 1.85; Women: 1.36	
Relative Risk for Hypertension from		
Overweight (BMI 25 kg/m^2^ to 29.9 kg/m^2^)	Men: 1.48; Women: 1.70	
Obesity (BMI 30+ kg/m^2^)	Men: 2.23; Women: 2.63	
Relative Risk for MI from		
Overweight (BMI 25 kg/m^2^ to 29.9 kg/m^2^)	1	
Obesity (BMI 30+ kg/m^2^)	1	
Relative Risk for CHD from		
Overweight (BMI 25 kg/m^2^ to 29.9 kg/m^2^)	1.43	
Obesity (BMI 30+ kg/m^2^)	1.58	
Relative Risk for Stroke from		
Overweight (BMI 25 kg/m^2^ to 29.9 kg/m^2^)	1	
Obesity (BMI 30+ kg/m^2^)	1	
Annual change in BMI (kg/m^2^) for populations age 30 to 49		Lewis et al. 2000[20]
White Male	0.23	
White Female	0.24	
Black Male	0.32	
Black Female	0.41	
Annual change in BMI (kg/m^2^) for populations age 50+		Botoseneanu and Liang 2011[21]
White Male	0.073	
White Female	0.073	
Black Male	0.020	
Black Female	0.020	

Abbreviations: BMI = Body Mass Index. Notes: Model parameters for relative risk related to obesity and annual change in obesity were derived from the literature. Sources of each parameter are listed in the table.

### Lifetime incidence of CKD

The lifetime incidence of CKD was estimated for persons with normal weight, overweight, and obesity at baseline. We conducted simulations overall and by baseline age (30 to 49, 50 to 64, and ≥65 years). Simulations were conducted with cohorts from NHANES 1999–2010, using a person’s starting age, sex, race/ethnicity, eGFR, albuminuria status (normal, moderately increased albuminuria, or severely increased albuminuria), diabetes status, hypertension status, cardiovascular disease status, and BMI. eGFR was computed using the CKD-Epi equation.[22] Because CKD staging is based on persistent albuminuria and NHANES only includes one observation of albuminuria per person, we adjusted observed moderately increased albuminuria using an algorithm proposed by Coresh et al.[23]

We simulated each person’s progression through the model until death or age 90. The starting year for the simulation is 2010. During each year, a person can develop diabetes, hypertension, overweight, obesity, or albuminuria. We estimated the lifetime risk of CKD as the probability of reaching any stage of CKD (stages 1–5). We also estimated lifetime risk of reaching each stage by death or age 90. We used bootstrapping to estimate 95% confidence intervals, simulating the lifetime risk 100 times. From this sample, we used the 2.5 and 97.5 percentile results to calculate confidence intervals.

### Sensitivity analyses

To test the sensitivity of our results and conclusions to the choice of parameters for risks associated with obesity, we conducted six one-way sensitivity analyses by varying key model parameters by ±25%: the relative risk of diabetes for obesity, the relative risk of hypertension for obesity, and the annual change in eGFR for persons with obesity. For each test, we examined the difference in lifetime risk of CKD between persons with obesity and normal weight at baseline. These parameters relate to the impact of obesity on CKD progression and other risk factors for CKD progression, so varying them tests the sensitivity of results to these risks.

## Results

**Fig 1:**
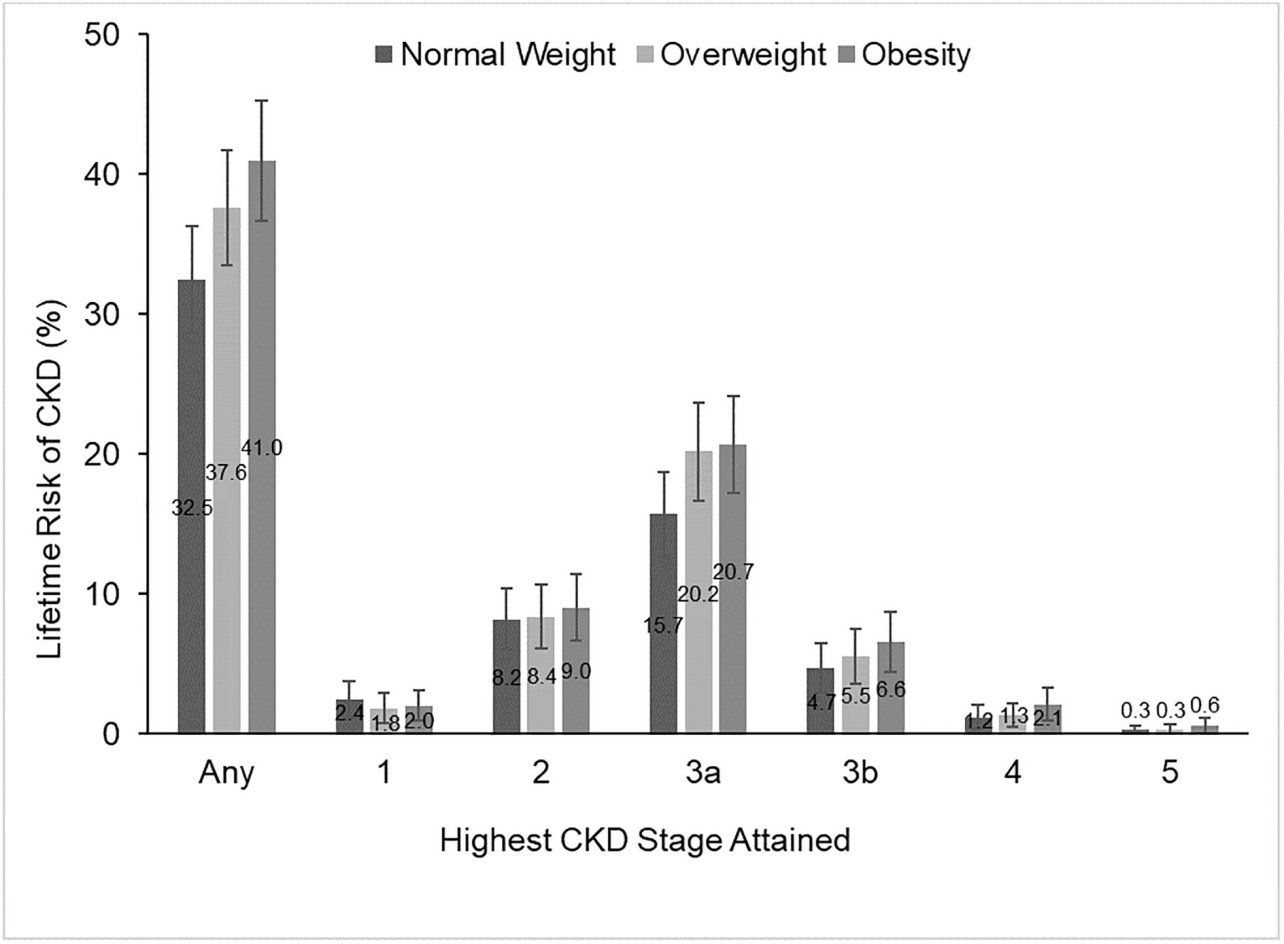
Lifetime risk of CKD, by baseline BMI category and highest CKD stage attained. The difference in lifetime risk or any CKD between persons with obesity and normal weight was statistically significant at the 1% level. No other differences were statistically significant. Abbreviations: CKD = Chronic Kidney Disease, BMI = Body Mass Index.

Fig 1 presents results of the lifetime risk of any-stage CKD and the highest CKD stage reached for persons with normal weight, overweight, or obesity at baseline. Lifetime risk of any-stage CKD was 32.5% (95% CI 28.6%–36.3%) for normal weight, 37.6% (95% CI 33.5%–41.7%) for overweight, and 41.0% (95% CI 36.7%–45.3%) for persons with obesity at baseline. The difference in lifetime risk of CKD between persons with normal weight and obesity at baseline was statistically significant (p<0.01). Persons with baseline obesity had a higher estimate of lifetime risk of CKD stages 4 (2.1%, 95% CI 0.9%–3.3%) and 5 (0.6%, 95% CI 0.0% -1.1%) than persons with normal weight (stage 4: 1.2%, 95% CI 0.3%–2.0%; stage 5: 0.3%, 95% CI 0.0%–0.7%). While these differences are relatively large (75% higher for stage 4 and 100% higher for stage 5), they were not statistically significant due to the challenge of getting sufficient precision with such small initial percentages (stage 4: p = 0.08; stage 5: p = 0.23).

**Fig 2:**
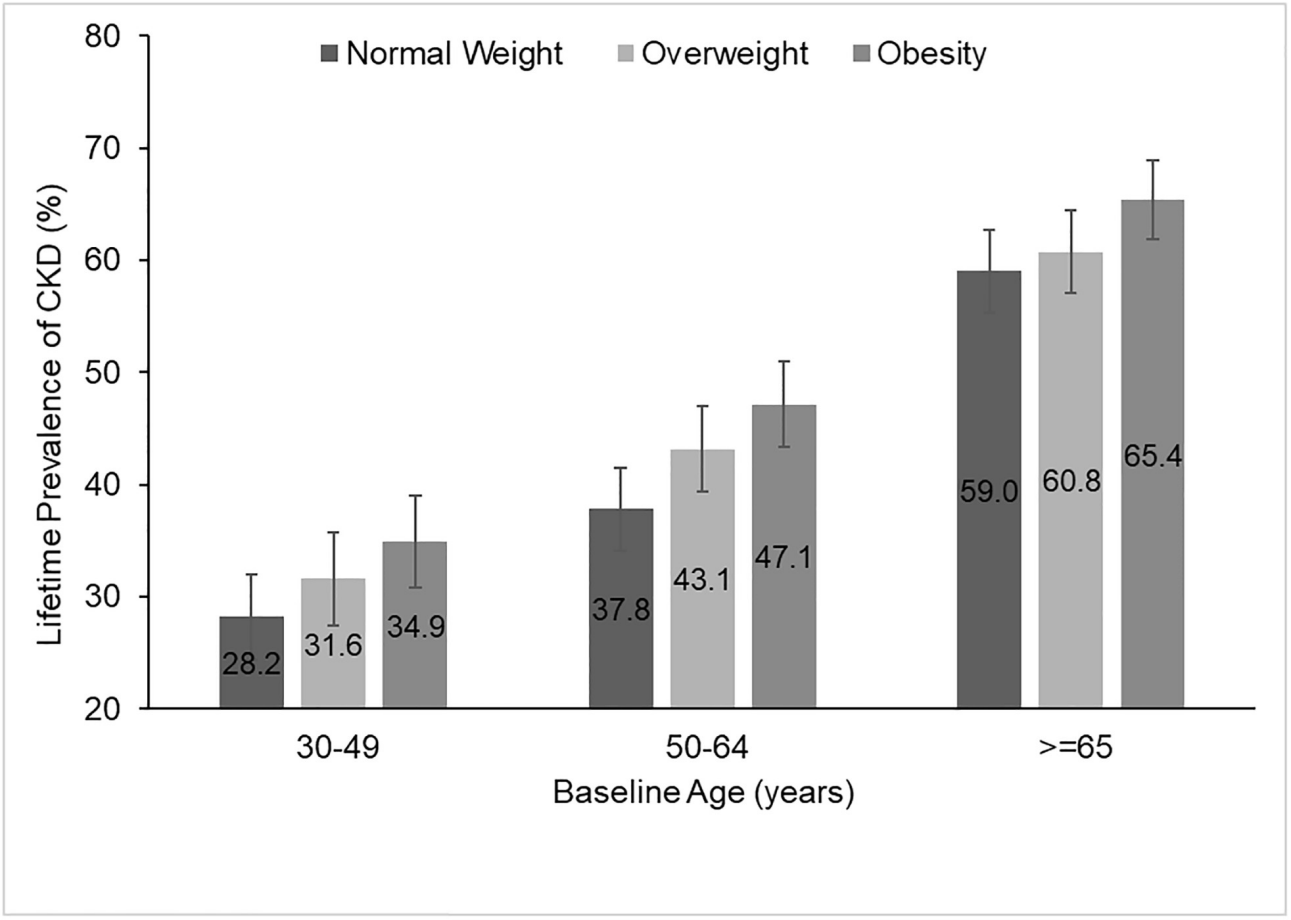
Lifetime risk of any CKD, by baseline BMI category and age. The difference in lifetime risk of any CKD between persons age 50–64 with obesity and normal weight was statistically significant at the 1% level. No other differences were statistically significant. Abbreviations: CKD = Chronic Kidney Disease, BMI = Body Mass Index.

Fig 2 presents the lifetime risk of any-stage CKD by baseline BMI and age categories. Lifetime risk of CKD increased with age, and within age groups estimates increased with BMI category. Obesity was significantly associated with a higher risk of CKD only among persons 50–64 years old [47.1%, 95% CI 43.3%–51.0% with obesity vs. 37.8%, 95% CI 34.1%–41.5% with normal weight, p<0.01).

**Fig 3:**
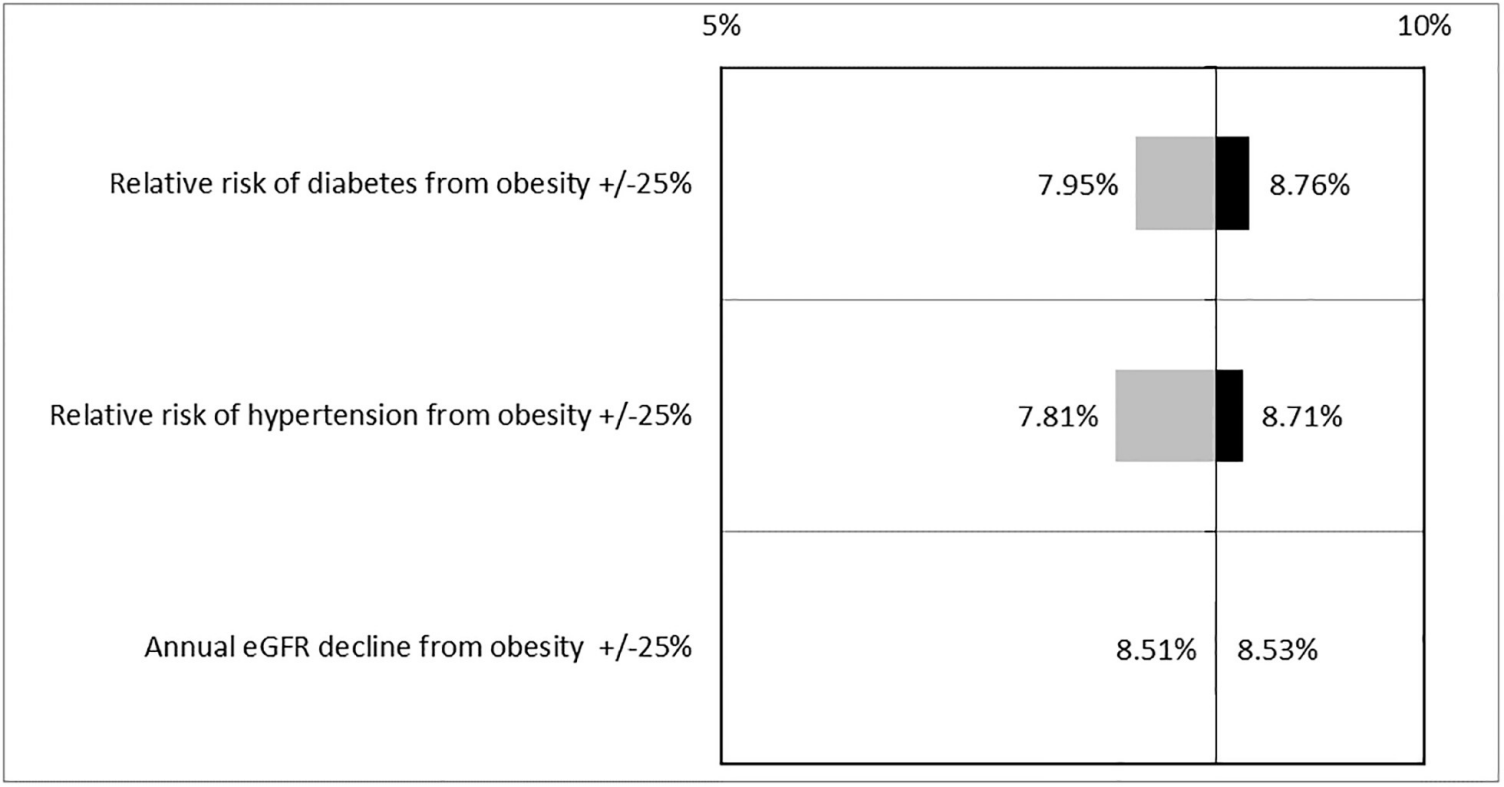
One-way sensitivity analysis of +/- 25% changes in key parameters on the difference in lifetime risk of any stage CKD between persons with obesity and persons with normal weight. In the main analysis, the difference in lifetime risk between persons with normal weight and obesity was 8.5%. Bars to the left of this value in the figure show the change in results if parameters are decreased by 25% and bars to the right of this value show the change in results if the parameters are increased by 25%. Abbreviations: eGFR = estimated glomerular filtration rate.

Fig 3 shows the results of one-way sensitivity analysis for 25% changes in parameter estimates on the difference in lifetime risk of CKD between persons with obesity and normal weight at baseline. These parameters relate to the impact of obesity on CKD progression and other risk factors for CKD progression. Varying the parameters for the impact of obesity on hypertension and diabetes had the greatest impact on the estimated difference in lifetime risk of CKD between persons with obesity and normal weight at baseline. However, differences were relatively modest, demonstrating that results are not sensitive to the assumptions of these model parameters. Model parameters would have to differ by much more than the 25% tested here to substantially change results.

## Discussion

This study presents the first simulation modeling of the impact of obesity on lifetime risk of CKD. This is an important innovation, because simulation modeling is essential to evaluate a time period sufficient for estimating lifetime risk. We found that compared with normal weight, obesity significantly increases the lifetime risk of any-stage CKD. Obesity was also found to be associated with lifetime risk of CKD stages 4 and 5. In the 50–64 age group, the lifetime risk of any-stage CKD was 9.3 percentage points higher in those with obesity than in those with normal weight. This is likely because persons in the younger age group with normal weight at baseline have a higher likelihood of developing obesity over time, mitigating differences with the group of persons with obesity at baseline, and because persons in the older age group have less time to develop CKD. While results were not statistically significant at the 5% level, the lifetime risk of CKD stages 4 and 5 could be doubled for those with baseline obesity, which might be translated to future high health and cost burden. For example, the estimated excess lifetime risk of stage 5 CKD would increase the cases of stage 5 CKD by approximately 350,000 at the current rate of adult obesity (36.5%),[24] which could be expected to cost approximately $21 billion annually.[25]

The literature indicates that this association may be driven either through obesity’s impact on diabetes, hypertension, and cardiovascular disease,[4–8] or directly through obesity’s impact on GFR.^9^ However, some studies have found that after controlling for diabetes and hypertension the direct effect of obesity on GFR is minimal.[8,10–13] We tested both a direct effect of obesity on CKD and an indirect effect through diabetes and hypertension. The results from this study of long-term measures of CKD are in keeping with the literature on short-term and intermediate measures. Namely, there was a significant association between obesity and lifetime risk of CKD, but sensitivity analysis showed that even when the direct effect of obesity on CKD is significantly higher than in the literature the relationship is driven almost exclusively by the effect on diabetes and hypertension.

This study is limited by the availability of parameters in the scientific literature. Although model parameters were based on current scientific literature, they may be imperfect or may omit additional unknown factors. For example, despite much research being done on the causal pathways from obesity to CKD, diabetes, and hypertension, much remains uncertain about the true impact. As this research develops, the model can be updated to include any new evidence that is generated.

The results of this study indicate that obesity may have an impact on the lifetime risk of CKD. These differences may be large and may have important implications for the expected future healthcare costs associated with CKD from rising rates of obesity. Results further suggest that obesity prevention may have important additional health and cost benefits through reducing the risk of CKD. This is important information for policymakers seeking to set priorities and targets for CKD prevention and treatment. The knowledge of factors that do and do not impact the burden of CKD allows policymakers to direct prevention efforts in the most impactful manner.

## Supporting information

S1 File CKD health policy model technical report. This file contains the detailed information about model construction, data, and parameters that were used to generate the results presented in this manuscript.
(DOC)
