# Supplementary material for: Modeling the impact of obesity on the lifetime risk of chronic kidney disease in the United States using updated estimates of GFR progression from the CRIC study
Source: PLoS One. 2018 Oct 19;13(10):e0205530. doi: 10.1371/journal.pone.0205530 (PMC6195263; doi:10.1371/journal.pone.0205530)
Supplement: S1 File — This file contains the detailed information about model construction, data, and parameters that were used to generate the results presented in this manuscript. (DOC) [file pone.0205530.s001.doc]

CKD Health Policy Model

Technical Report

December 2015

Thomas J. Hoerger, PhD,^1^ John S. Wittenborn, BS,^1^ Xiaohou Zhou, PhD,^2^ Meda E. Pavkov, MD, PhD,^2^ Nilka R. Burrows, MPH,^2^ Paul Eggers, PhD,^3^ Regina Jordan, MPH,^2^ Sharon Saydah, and Desmond E. Williams, MD, PhD^2^ for the CDC CKD Initiative

^1^RTI International

^2^Centers for Disease Control and Prevention

^3^National Institute of Diabetes and Digestive and Kidney Diseases

Send all correspondence to:

Thomas J. Hoerger

RTI International

3040 Cornwallis Road

P.O. Box 12194

Research Triangle Park, NC 27709

Voice: (919) 541-7146

Fax: (919) 541-6683

E-mail: tjh@rti.org

Contents

Section Page

[**1.** **Introduction 1-1**](#__RefHeading___Toc313013046)

[1.1 Project Objectives 1-1](#__RefHeading___Toc313013047)

[1.2 Background 1-2](#__RefHeading___Toc313013048)

[2. Model Overview 2-1](#__RefHeading___Toc313013049)

[3. Chronic Kidney Disease and Stages 3-1](#__RefHeading___Toc313013050)

[3.1 Kidney Damage 3-1](#__RefHeading___Toc313013051)

[3.2 Glomerular Filtration Rate 3-4](#__RefHeading___Toc313013052)

[4. Risk Factors 4-1](#__RefHeading___Toc313013053)

[4.1 Diabetes 4-1](#__RefHeading___Toc313013054)

[4.2 Systolic Blood Pressure and Hypertension 4-1](#__RefHeading___Toc313013055)

[4.3 Cholesterol 4-2](#__RefHeading___Toc313013056)

[4.4 Smoking Status 4-3](#__RefHeading___Toc313013057)

[4.5 Left Ventricular Hypertrophy 4-3](#__RefHeading___Toc313013058)

[5. Complications 5-1](#__RefHeading___Toc313013059)

[5.1 Cardiovascular Disease 5-2](#__RefHeading___Toc313013060)

[5.2 Coronary Heart Disease and Myocardial Infarction 5-2](#__RefHeading___Toc313013061)

[5.3 Stroke 5-3](#__RefHeading___Toc313013062)

[6. Mortality 6-1](#__RefHeading___Toc313013063)

[6.1 Non-CVD Deaths 6-1](#__RefHeading___Toc313013064)

[6.2 CVD Deaths 6-2](#__RefHeading___Toc313013065)

[6.3 Stage 5 and ESRD Mortality 6-3](#__RefHeading___Toc313013066)

[7. Costs and Utility Values 7-1](#__RefHeading___Toc313013067)

[7.1 Early CKD Stage Costs 7-1](#__RefHeading___Toc313013068)

[7.2 ESRD Stage Costs 7-2](#__RefHeading___Toc313013069)

[7.3 Effectiveness Measures 7-3](#__RefHeading___Toc313013070)

[8. Medical Care and Interventions 8-1](#__RefHeading___Toc313013071)

[8.1 Integration of Hypothetical Treatment Scenarios 8-1](#__RefHeading___Toc313013072)

[8.2 Screening and Treatment Costs 8-4](#__RefHeading___Toc313013073)

[9. Race-specific Progression Calibration 9-1](#__RefHeading___Toc313013074)

[9.1 African American CKD Progression Risk Factors 9-1](#__RefHeading___Toc313013075)

[9.2 Other Potential Factors in CKD Progression among African Americans 9-4](#__RefHeading___Toc313013076)

[9.3 Calibration of GFR to Match African American ESRD Incidence Rates 9-6](#__RefHeading___Toc313013077)

[10. Model Validation 10-1](#__RefHeading___Toc313013078)

[10.1 Validation Process 10-1](#__RefHeading___Toc313013079)

[10.2 Parameterization Testing and Internal Validation 10-1](#__RefHeading___Toc313013080)

[10.3 CKD Progression Validation 10-3](#__RefHeading___Toc313013081)

[References R-1](#__RefHeading___Toc313013082)

**Appendix**

A: Data Inputs A-1

Figures

Number Page

2-1. Simplified Decision Analysis Tree [2-2](#__RefHeading___Toc313013777)

8-1. Schematic of Screen and Treat Intervention [8-4](#__RefHeading___Toc313013778)

Tables

Number Page

[3-1. K/DOQI CKD Stage Definitions 3-1](#__RefHeading___Toc313013858)

[3-2a. Prevalence of Persistent Micro- and Macroalbuminuria 3-3](#__RefHeading___Toc313013859)

[3-2b. Prevalence of Persistent Micro- and Macroalbuminuria 3-3](#__RefHeading___Toc313013860)

[3-3. Annual GFR Decrements 3-5](#__RefHeading___Toc313013861)

[4-1. Smoking Prevalence 4-3](#__RefHeading___Toc313013862)

[5-1. CKD Stage CVD Multipliers 5-2](#__RefHeading___Toc313013863)

[6-1. Mortality Data Table from Go et al. (2004) 6-1](#__RefHeading___Toc313013864)

[6-2. Relative Rates of CKD Mortality 6-2](#__RefHeading___Toc313013865)

[6-3. Excess Mortality Due to Myocardial Infarction 6-3](#__RefHeading___Toc313013866)

[7-1. Annual Costs of CKD and Complications 7-2](#__RefHeading___Toc313013867)

[7-2. Utility Values 7-3](#__RefHeading___Toc313013868)

[8-1. Selected Model Parameters 8-2](#__RefHeading___Toc313013869)

[8-2. Literature Review of Effect of ACE Inhibitor Use on GFR Progression 8-4](#__RefHeading___Toc313013870)

[8-3. Aggregated Intervention Costs 8-6](#__RefHeading___Toc313013871)

[9-1. Impact of Race-Specific Blood Pressure Values on CKD Progression among African Americans 9-2](#__RefHeading___Toc313013872)

[9-2. Impact of Race-Specific Diabetes Prevalence and Incidence on CKD Progression among African Americans 9-3](#__RefHeading___Toc313013873)

[9-3. Impact of Race-Specific Microalbuminuria Incidence and Transition to Macroalbuminuria on CKD Progression among African Americans 9-4](#__RefHeading___Toc313013874)

[9-4. Impact of No Preventive Medical Care on CKD Progression among African Americans 9-4](#__RefHeading___Toc313013875)

[9-5. Impact of Immediate Entry to ESRD upon Initiation of Stage 5 on CKD Progression among African Americans 9-5](#__RefHeading___Toc313013876)

[9-6. Impact of Race-Specific GFR Distributions on CKD Progression among African Americans 9-6](#__RefHeading___Toc313013877)

[9-7. Impact of Race-Specific GFR Multipliers on CKD Progression among African Americans 9-6](#__RefHeading___Toc313013878)

[10-1. Internal Validation Results, SBP in Non-CKD Men 10-2](#__RefHeading___Toc313013879)

[10-2. Internal Validation Results, Total Cholesterol in Men 10-2](#__RefHeading___Toc313013880)

[10-3. Internal Validation Results, HDL Cholesterol 10-3](#__RefHeading___Toc313013881)

[10-4. Internal Validation of Albuminuria Prevalence 10-4](#__RefHeading___Toc313013882)

[10-5. External Validation of CKD Stage Prevalence Rates 10-4](#__RefHeading___Toc313013883)

[10-6. External Validation of Stage 5 Incidence, CKD20081105 10-5](#__RefHeading___Toc313013884)

[10-7. Selected Model Output 10-6](#__RefHeading___Toc313013885)

# Introduction

This technical supplement summarizes the design and construction of the cost-effectiveness model used in the manuscript “Chronic Kidney Disease Progression and Screening Cost-Effectiveness among African Americans.” This model was developed by RTI International, under contract with the Centers for Disease Control and Prevention (CDC), Division of Diabetes Translation.

## 1.1 Project Objectives

The successful implementation of primary prevention, delay, and treatment interventions for chronic kidney disease (CKD) requires innovative strategies to address the scientific, program, and policy issues associated with the interventions. Some of the scientific evidence for the efficacy of interventions is known, but more is being developed. According to some sources, screening may be underutilized and clinical care may be suboptimal for CKD.

Common tests for early kidney damage include measuring urine albumin and creatinine. Current recommendations call for annual screening for microalbuminuria and macroalbuminuria among diabetes patients (National Kidney Foundation, 2007). Simple tests may also be cost-effective for persons with hypertension or other high-risk populations. A decreased glomerular filtration rate (GFR)—an indicator of kidney function estimated from serum creatinine—is associated with worsening kidney disease and increased risk of death, cardiovascular events, and hospitalization. Preventive care practices include screening for kidney diseases, monitoring and controlling blood pressure, using angiotensin-converting enzyme inhibitors and other medicines for diabetic and nondiabetic nephropathies, maintaining glycemic control in persons with diabetes, and maintaining low-protein diets.

To improve public health applications for prevention and treatment, a cost-effectiveness model is necessary to evaluate both existing and future interventions for CKD. The purpose of this project is to produce a model to accurately reflect the early-stage incidence and progression of CKD in a U.S. population cohort. This natural history model will facilitate the integration of screening and medical treatment, which will improve the understanding of the cost-effectiveness of interventions intended to mitigate the burden of CKD. The primary benefits of interventions are

- avoiding medical costs and quality-adjusted life year (QALY) losses incurred by those who suffer from advanced CKD;
- substituting less expensive early-stage therapies for more expensive late-stage therapies; and
- avoiding medical costs and QALY losses associated with other diseases and complications that may be adversely affected by CKD progression, such as cardiovascular disease (CVD) and coronary heart disease (CHD).

The model will facilitate assessment of these outcomes by linking costs, mortality, and utility values to the progression of CKD and its complications and allow the study of interventions that influence the natural history of the disease.

## 1.2 Background

CDC’s National Center for Chronic Disease Prevention and Health Promotion (NCCDPHP) seeks to enable persons with CKD to lead long, healthy, and satisfying lives by preventing death and disability. To accomplish this goal in the face of escalating health care costs, NCCDPHP investigates and assesses practical interventions for controlling and preventing CKD. Among its approaches to CKD is the construction of a cost-effectiveness model. The model will assess upstream prevention strategies that reduce the burden of CKD and treatment interventions that delay progression and reduce comorbidities.

CKD is a major cause of mortality, morbidity, and cost. When considering CKD, end-stage renal disease (ESRD) immediately comes to mind, as it is an easily defined condition that causes great mortality and morbidity and incurs great costs. However, a growing body of evidence demonstrates that pre-ESRD CKD can also cause significant morbidity and cost, both directly and by exacerbating other chronic conditions such as CVD (Go et al., 2004; Smith et al., 2007; Weiner et al., 2004). While 506,000 persons in the United States have ESRD, an estimated 26 million have early stage CKD (Coresh et al., 2007; USRDS, 2008a).

The CKD burden is differentiated by race/ethnicity. An estimated 2.5% of white men and 1.8% of white women are at risk for ESRD in their lifetimes compared with 7.3% of African American men and 7.8% of African American women (Kiberd and Clase, 2002).

In 2001, the total expenditures (Medicare and private payers) for kidney disease exceeded $22 billion. Persons with ESRD constitute 1% of the Medicare population but consume 6.4% of Medicare health care expenditures. Even more alarming, the total expenditures for CKD patients were approximately twice those of ESRD patients (USRDS, 2008b, c).

# Model Overview

The chronic kidney disease (CKD) model is a discrete state simulation model programmed in TreeAge Pro 2008 using the software’s Markov Monte Carlo microsimulation functions. The model consists of seven mutually exclusive states representing CKD status, with annual transitions between states governed by two disease variables: glomerular filtration rate (GFR) and proteinuria. The model employs tracker variables to govern risk factors and complications. The model is intended to accurately depict the incidence and progression of CKD in a cohort of simulated individuals (agents) aged 30 until age 90 or death. The model will capture each agent’s relevant medical outcomes, costs, and utility measures associated with kidney disease and its complications from any specified age until death. This approach allows the model to generate predicted disease, risk factor, and complication status for every age while allowing the model the flexibility to specify any age as the baseline age for interventions and analysis. The microsimulation structure of the model was selected to allow an accurate and realistic depiction of disease incidence, progression, and treatment. Despite the use of mutually exclusive disease states, this approach differs from a Markov model structure in that it allows parameters to be stochastically distributed across the population, allows individual agent characteristics and history to influence future events, and allows nonmutually exclusive risk factors and complications.

The model has seven primary states: normal, dead, and five states representing the five Kidney Disease Outcomes Quality Initiative (K/DOQI) stages of CKD (Figure 2-1). Progression from normal to and through the K/DOQI states is governed by patients’ simulated GFR and proteinuria status (Levey et al., 2003). Mortality is assigned based on annual background rates, CKD stage-specific non-cardiovascular disease (CVD) rates, CVD rates determined by myocardial infarction (MI) and stroke events, and end-stage renal disease (ESRD) rates. Risk factors and medical events are simulated annually based on probability functions. Model risk factors include diabetes status, systolic blood pressure and hypertension, left ventricular hypertrophy (LVH), total and high-density lipoprotein (HDL) cholesterol, and smoking status. Discrete medical events that are tracked include stroke and coronary heart disease (CHD), including MI and angina. Individual-level risk factors and events are simulated for all stages except stage 5, which is modeled by assigning mean population cost, mortality, and utility values for persons with ESRD. Focusing on early disease stages allows the model to be used to assess the cost-effectiveness of various prevention, early detection, and treatment interventions.

Parameterization of the model was accomplished based on an in-depth review of the literature, consultation with a CKD expert panel, and derivation using data from the National Health and Nutrition Examination Survey and Medicare claims. We validated the model according to recommended standards outlined by the International Society for Pharmacoeconomics and Outcomes Research Task Force (Weinstein et al., 2003).

Figure 2-1. Simplified Decision Analysis Tree

# Chronic Kidney Disease and Stages

The simulation model assigns agents annually to one of seven states: normal (no chronic kidney disease [CKD]), dead, or one of five Kidney Disease Outcomes Quality Initiative (K/DOQI) stages. The stages follow the definitions included in the National Kidney Foundation K/DOQI guidelines and are based on kidney damage and/or specified measures of glomerular filtration rate (GFR) (Table 3-1). Kidney damage is defined as structural or functional abnormalities of the kidney, including pathological abnormalities or markers of damage such as imaging abnormalities or abnormalities in the composition of the blood or urine. In practice, kidney damage is typically indicated by the presence of albuminuria. GFR is a measure of the filtering functionality of the kidney and declines in a relatively linear pattern with age. A notable feature of the K/DOQI guidelines is that kidney damage is required for assignment to stages 1 and 2, whereas stages 3, 4, and 5 are defined solely based on GFR. Many patients in stage 3 and after do not in fact have kidney damage, meaning it is possible for an individual who never gets kidney damage to progress from normal directly to stage 3. For the purposes of our model, we assume that persons entering stage 5 will, on average, require 1 year in stage 5 before the initiation of ESRD.

Table 3-1. K/DOQI CKD Stage Definitions

| State | Kidney Damage | GFR |
| --- | --- | --- |
| Normal | No | 60+ |
| 1 | Yes | 90+ |
| 2 | Yes | 60–89 |
| 3 | Yes or No | 30–59 |
| 4 | Yes or No | 15–30 |
| 5 | Yes or No | <15 |

## 3.1 Kidney Damage

Small amounts of protein are excreted in the urine of healthy individuals; however, the presence of increased protein, or proteinuria, may indicate the presence of kidney disease. The increased excretion of the protein albumin may indicate CKD due to diabetes, glomerular disease, or hypertension—the most common causes of CKD. Consequently, the K/DOQI guidelines suggest screening for CKD by testing for elevated levels of urine albumin using the quantitative test of a urine albumin-creatinine ratio (ACR), which can indicate micro- or macroalbuminuria, or a more basic urine protein dipstick test.

Kidney damage is an important aspect of identifying CKD. We define kidney damage as persistent microalbuminuria and macroalbuminuria. Micro- and macroalbuminuria are defined using urine albumin-creatinine values and thus focus on the specific protein, albumin. For purposes of this report, all references to microalbuminuria are considered persistent. Micro- or macroalbuminuria is used to assign individuals to stage 1 or 2 CKD according to the K/DOQI guidelines. Whereas the K/DOQI definitions include diagnoses of kidney damage through imaging or other diagnostic tests, in our potential data sources, kidney damage is defined only through detection of abnormal ACRs. Individuals with microalbuminuria can progress to macroalbuminuria, which is used to assign individuals a faster GFR progression rate and in the analysis of screening. Evidence suggests that macroalbuminuria is associated with accelerated reductions in GFR and thus will impact CKD progression beyond stages 1 and 2.

Prevalent microalbuminuria is assigned at model initiation, with subsequent annual incidence rates (Tables A‑2a, A-2b). Agents with microalbuminuria face an annual probability of progression from micro- to macroalbuminuria (Table A‑3). Using combined National Health and Nutrition Examination Survey (NHANES) data from 1999–2006, we analyzed the prevalence of albuminuria in the adult population by calculating the ACR using urine albumin and creatinine data provided in the lab data. We used a single cutoff value to define micro- and macroalbuminuria, using the definition reported in Coresh et al. (2007). Microalbuminuria was defined as an ACR of 30 to 299 mg/g, whereas macroalbuminuria was defined as an ACR of 300 mg/g or higher. The latest NHANES data do not include follow-up ACR measures, so persistent microalbuminuria, which is required for a diagnosis of stage 1 or 2 CKD, could not be identified. We used a coefficient of 0.509 for individuals with a GFR greater than 90 ml/min per 1.73 m^2^, 0.75 for those with a GFR between 60 and 89 ml/min per 1.73 m^2^, and 1 for those with a GFR less than 60, all from Coresh et al. (2007), to approximate the proportion of all microalbuminuria that is persistent. We assume that 100% of observed macroalbuminuria cases are persistent. The use of persistence values leads to lower estimates of microalbuminuria than those provided in Saydah et al. (2007), which did not include persistence. We estimated the prevalence of micro-, macro-, and total albuminuria (either micro- or macroalbuminuria) separately for men and women by age groups (ages 30 to 49, 50 to 64, 65 or older). We further estimated the prevalence for individuals with neither hypertension nor diabetes, individuals with just hypertension, and individuals with diabetes (with or without hypertension). We defined hypertension as having systolic blood pressure greater than or equal to 140 mm Hg, diastolic blood pressure greater than or equal to 90 mm Hg, or reporting a diagnosis of hypertension. Diabetes was defined only by self-report of a diagnosis. Table 3‑2a shows prevalence rates for persistent micro- and macroalbuminuria from 1999–2004 NHANES data. Table 3‑2b shows prevalence rates for African Americans and all other races (non-African Americans) in 1999–2006 NHANES data.

Table 3-2a. Prevalence of Persistent Micro- and Macroalbuminuria

| **Albuminuria Type** | **Women Neither** | **Men Neither** | **Hypertension Women** | **Hypertension Men** | **Diabetes Women** | **Diabetes Men** |
| --- | --- | --- | --- | --- | --- | --- |
| Persistent microalbuminuria |  |  |  |  |  |  |
| Ages 30–49 | 2.4% | 2.0% | 6.5% | 3.9% | 13.6% | 18.3% |
| Ages 50–65 | 4.3% | 2.7% | 6.2% | 6.3% | 11.0% | 17.9% |
| Ages 65+ | 7.6% | 9.3% | 14.4% | 15.9% | 21.1% | 22.6% |
| Macroalbuminuria |  |  |  |  |  |  |
| Ages 30–49 | 0.2% | 0.2% | 0.6% | 1.5% | 1.5% | 3.3% |
| Ages 50–65 | 0.2% | 0.3% | 0.8% | 1.8% | 7.8% | 7.6% |
| Ages 65+ | 0.1% | 1.1% | 2.4% | 3.9% | 7.4% | 13.0% |
| Total albuminuria |  |  |  |  |  |  |
| Ages 30–49 | 2.6% | 2.2% | 7.1% | 5.4% | 15.1% | 21.6% |
| Ages 50–65 | 4.5% | 3.0% | 7.0% | 8.1% | 18.8% | 25.5% |
| Ages 65+ | 7.7% | 10.4% | 16.8% | 19.8% | 28.5% | 35.6% |
| Mean age |  |  |  |  |  |  |
| Ages 30–49 | 39.3 | 39.1 | 41.6 | 41.0 | 42.3 | 42.1 |
| Ages 50–65 | 55.4 | 55.3 | 56.8 | 56.3 | 56.6 | 56.8 |
| Ages 65+ | 72.5 | 72.7 | 74.9 | 73.9 | 73.8 | 71.9 |

Table 3-2b. Prevalence of Persistent Micro- and Macroalbuminuria, By Race

|  | **Microalbuminuria Prevalence** | | |  | **Macroalbuminuria Prevalence** | | |
| --- | --- | --- | --- | --- | --- | --- | --- |
| **Race** | **20-49** | **50-64** | **65-90** |  | **20-49** | **50-64** | **65-90** |
| African American | 0.069 | 0.143 | 0.190 |  | 0.021 | 0.041 | 0.069 |
| Non-African American | 0.048 | 0.084 | 0.171 |  | 0.003 | 0.014 | 0.032 |

The model assumes that patients have microalbuminuria upon incidence of damage and then may transition to macroalbuminuria in subsequent years based on annual transition probabilities. We identified age-specific prevalence rates for persistent micro- and macroalbuminuria based on NHANES data for six cohorts consisting of men and women with hypertension, men and women with diabetes (with or without hypertension), and men and women with neither diabetes nor hypertension. For agents with diabetes, the annual incidence of microalbuminuria is 2% and the annual probability of progression to macroalbuminuria is 2.84% (Adler et al., 2003). We were unable to identify suitable microalbuminuria incidence rates for persons without diabetes. For cohorts with only hypertension or no diabetes or hypertension, we fit a second-degree polynomial to the total of persistent microalbuminuria and macroalbuminuria to yield a smoothed prevalence function that increased with age. Based on this function, we calculated the annual incidence of damage for men and women. For persons with hypertension only, we identified an annual transition from micro to macro as 1.47% (Mann et al., 2003). We were unable to identify a suitable micro-to-macro transition probability in the literature for persons with neither diabetes nor hypertension. Therefore, we identified rates using linear programming to solve for the micro-to-macro transition probabilities that predicted the same prevalence of macroalbuminuria as found in NHANES data.

We found that African Americans experience higher prevalence of micro- and macroalbuminuria at all ages. We differentiated the microalbuminuria incidence and transition to macroalbuminuria by race to account for higher prevalence observed in African Americans. We solved for coefficients of microalbuminuria incidence and transition to macroalbuminuria to most closely match the race-specific prevalence rates observed in NHANES for African Americans and all others that retained the overall prevalence rates.

## 3.2 Glomerular Filtration Rate

GFR serves as the other primary variable for tracking progression of CKD between stages. The validated model employs a process in which agents are assigned a normally distributed initial GFR and then experience annual decrements in GFR based on certain risk factors. Patients are randomly assigned an age 30 GFR starting value based on a normal distribution with a mean of 101.9409 ml/min per 1.73 m^2^ and a standard deviation of 19.313. The mean and standard deviations are based on NHANES III data for individuals aged 25 to 35, where GFR was estimated using the simplified Modification of Diet in Renal Disease (MDRD) equation for standardized serum creatinine values developed by Levey et al. (2006). We estimated GFR in the NHANES data with this new equation: GFR = 175 X (standardized serum creatinine)^−1.154^ X (age)^−0.203^ X 0.742 (if woman) X 1.212 (if black).

The annual decrement values are based on the baseline rates used by Boulware et al. (2003) and vary based on CKD stage and hypertension status (Table 3-3). We adjusted these rates to account for the observed decline in GFR with age in the most recent cross-sectional NHANES data (2003–2004) for persons without albuminuria. We found that this value was 0.653 ml/min per 1.73 m^2^ per year, whereas Boulware et al. had assumed a value of 1.0 ml/min per 1.73 m^2^ per year. Boulware et al. also assumed that progression was 10% faster than the 1.0 ml/min per 1.73 m^2^ baseline rates for individuals with (a) no diabetes, no hypertension, GFR > 60, and proteinuria; and (b) hypertension, GFR > 60, and no proteinuria. We have retained Boulware et al.’s assumption of a 10% increase for these cases but now apply it to the lower baseline rate of 0.653 ml/min per 1.73 m^2^.

Table 3-3. Annual GFR Decrements

| **Diabetes/Hypertension Status** | **GFR** | **Annual GFR Decrement** |
| --- | --- | --- |
| Neither |  |  |
| No proteinuria | ≥ 60 | 0.653 |
|  | < 60 | 0.653 |
| Proteinuria | ≥ 60 | 0.719 |
|  | < 60 | 4.2 |
| Hypertension |  |  |
| No proteinuria | ≥ 60 | 0.719 |
|  | < 60 | 1.4 |
| Proteinuria | ≥ 60 | 0.784 |
|  | < 60 | 3.9 |
| Diabetes |  |  |
| No proteinuria | ≥ 60 | 1.1 |
|  | < 60 | 2.8 |
| Proteinuria | ≥ 60 | 4.1 |
|  | < 60 | 5.2 |

The annual decrement methodology does not allow for any variability in GFR between individuals with identical CKD and risk factor status. This can yield unrealistic results because all persons are assumed to progress at the mean observed rates. We added variability to the annual baseline decrement values by applying a randomly assigned multiplicative coefficient when an individual develops microalbuminuria based on a symmetric triangular distribution with a min, mode, and max of 0, 1, and 2, respectively. We selected this range based in part on the MDRD results showing the range of observed GFR slopes and also to prevent the occurrence of positive GFR slopes (Hunsicker et al., 1997). Other things being equal, agents with a high random draw will progress more quickly than average, whereas agents with a low random draw will progress less quickly. For example, an agent may draw a coefficient value of 1.2 from the triangular distribution. If that agent has macroalbuminuria, normal GFR, and diabetes, the agent will have an annual GFR decrement of 4.1 x 1.2 = 4.92 ml/min per 1.73 m^2^. Later in life, that agent may have a GFR level of 40, in which case his or her annual decrement value would be 5.2 x 1.2 = 6.24 ml/min per 1.73 m^2^. The model includes an absolute minimum GFR level of 0.

GFR reduction rates have been observed to be greater among African Americans. Based on the GFR slope coefficient found for African Americans in the MDRD study (Hunsicker et al., 1997), we considered increasing the absolute value of the annual GFR change by an extra 1.5 ml/min per 1.73 m^2^ for African Americans. However, we currently do not include this race adjustment factor because its inclusion yielded poorer overall CKD stage prevalence results in external validation.

Based on feedback from an external panel, we considered replacing the MDRD-derived GFR values and reduction rates with values based on the Rule et al. equation (Rule et al., 2004). Using the Rule equation resulted in a higher baseline GFR value than the MDRD (123.8 versus 101.9), a smaller standard deviation (12.6 versus 19.3), and a larger annual decrement (0.89 versus 0.65). In practice, the larger decrement does not counteract the higher initial value and, when combined with the smaller deviation, resulted in significantly higher estimated GFR values under all model scenarios. Although using the Rule equation may have achieved more conservative results, we found that external validation results using the Rule equation were extremely poor, yielding about 1/5 as many cases of ESRD as USRDS data would indicate (USRDS, 2006a; Arias, 2006). In 2009, Snyder et al. published a revised process for standardizing serum creatinine values in NHANES for use with the Rule equation. Using this adjustment, the Rule equation yields an initial value of 110.1256 and an annual decrement of 0.848. Using this adjustment, the Rule GFR values are higher than MDRD until approximately age 75 and lower thereafter. However, external validation results using this equation still yielded too few cases of ESRD and thus we decided to keep the MDRD-derived values.

The MDRD equation includes a 1.21 coefficient for African Americans, which is factored into the overall GFR values calculated from NHANES data. In the original model, African Americans are assigned the same GFR parameters as the rest of the population. We considered the impact of calculating GFR parameters separately by race. Doing this, we find African Americans have higher initial GFR values (111.575) and a higher annual decrement (0.757). This increases ESRD incidence by 0.001 with a similar increase in CKD prevalence. However, when combined with other race-specific progression parameters, we found that these GFR parameters result in a substantial overestimate of CKD prevalence. Thus, we do not differentiate GFR parameters by race, other than the black-race coefficient in the MDRD equation.

In the future, a probability of acute kidney disease could be integrated into our model that would allow for sudden and drastic decreases in GFR. These decreases could result in large changes in CKD stages over short periods (e.g., a jump from normal to stage 5 in 1 year). Currently, however, we do not have sufficient data to calculate the annual probability of acute kidney disease occurring, nor do we have data on the distribution of GFR decrements associated with acute kidney failure.

# Risk Factors

Risk factors play an important role in chronic kidney disease (CKD) incidence, progression, and outcomes. Risk factors are defined in the context of this model as non-acute conditions that are assigned to patients without regard to CKD status. For the purposes of this report, we define risk factors as distinct from complications, which are directly impacted by CKD. For example, in the model, CKD directly impacts cardiovascular disease (CVD) outcomes, and thus CVD is considered a complication. In reality, risk factors such as diabetes, blood pressure, and left ventricular hypertrophy (LVH) are believed to have a synergistic effect with CKD progression; these risk factors may advance CKD, and CKD may advance these risk factors. However, it is beyond the scope of this analysis to interpret the mechanics of this relationship, and thus we must make assumptions about causal relationships in correlated events.

## 4.1 Diabetes

Diabetes is an important risk factor for CKD and CKD complications. Diabetes is assigned based on age 30 prevalence and annual incidence rates thereafter (Table A‑4). Prevalence and incidence rates are dependent on age, sex, and race/ethnicity but not on CKD status. Diabetes accelerates CKD progression by triggering higher annual glomerular filtration rate (GFR) decrements as well as higher incidence of microalbuminuria and faster progression to macroalbuminuria, which lead to higher rates of proteinuria. Diabetes also results in higher rates of complications, including CVD, coronary heart disease (CHD), stroke, and myocardial infarction (MI) incidence and mortality.

Diabetes prevalence is assigned at age 30 for all individuals based on prevalence data for diagnosed and undiagnosed diabetes prevalence by age, race/ethnicity, and sex from the National Health and Nutrition Examination Survey (NHANES) reported by Cowie et al. (2006). We base age 30 prevalence of diabetes on the prevalence of diagnosed diabetes found among 20- to 39-year-olds based on race and sex. Annual incidence of diabetes is based on predictive margins of incidence found by Geiss et al. (2006) who did not find significant differences in incidence by sex; thus, men and women of each race are assigned the same incidence.

## 4.2 Systolic Blood Pressure and Hypertension

We simulate each person’s systolic blood pressure (SBP) and assign hypertension if SBP is higher than 140. SBP and hypertension are important indicators for the CKD model. SBP is used directly as an input in the Framingham equations governing CHD/CVD events and mortality. Hypertension status results in increased rates of GFR reduction and increases the probability of developing kidney damage, and increases the transition rate from microalbuminuria to macroalbuminuria. SBP may increase upon CKD incidence.

We identified mean and percentile rank values of SBP by age, race/ethnicity, sex, and CKD grade status in NHANES III data (Table A‑5). Values were identified based on sex and age group (ages 30–44, 45–54, 55–64, 65–74, 75–84, 85+) for three populations: the total population, the population without CKD, and the population with CKD. We defined the CKD populations using the methods from Coresh et al. (2003). To identify the distribution of SBP values, we calculated the SBP at the 5th, 10th, 15th, 25th, 50th, 75th, 85th, 90th, and 95th percentiles. To identify age trends in SBP, we assumed that the percentile rank value for each age was equivalent to a longitudinal sample. The data revealed that the distribution of SBP values increased with age and was skewed toward higher values. This results in SBP diverging over time with higher SBP values growing at a high rate. We fit a probability density function to the initial values of SBP and then determined a polynomial function for SBP slope by age, sex, and CKD status. We simulated SBP values for each person over the course of their lives by assigning each patient to a percentile rank of possible SBP values. Higher initial SBP is correlated with a faster increase in SBP with age. Thus, the patients’ percentile rank determines both their initial SBP value and subsequent slope. We also found that patients with CKD exhibited higher overall SBP. We modeled this relationship by solving for two separate SBP constant functions, one for CKD patients and one for non-CKD patients. Upon incidence of CKD, agents are assigned the CKD population SBP constant.

SBP is varied based on race/ethnicity by altering the SBP constant value. African Americans and Hispanics generally had higher mean SBP and greater variability in SBP distributions than whites. There was no clear correlation between these effects and age. We modeled the variability of SBP by race/ethnicity by adding two terms to the SBP constant equation, an increase in the constant value and an additional linear slope as a function of percentile rank. Any patient whose SBP exceeds 140 is assigned positive hypertension status.

## 4.3 Cholesterol

Total cholesterol and HDL cholesterol are parameters used in the Framingham equations that the model uses to assign CVD/CHD events and mortality. The model simulates individuals’ total cholesterol based on age and sex and HDL cholesterol based on sex. Data used to specify cholesterol levels come from the third National Institutes of Health (NIH) cholesterol education expert panel report (NCEP) that shows cholesterol levels by age at different percentile levels as calculated from NHANES III (Table A‑6). Total cholesterol illustrates an age effect by increasing with age until decreasing in the oldest age category. NHANES is a cross-sectional sample, so we do not know if this reduction is due to survivorship selection, but allowing a decrease in total cholesterol will yield more conservative results. HDL cholesterol shows no significant variation with age, so HDL levels are assigned as a constant value only.

Total cholesterol levels are simulated separately for men and women in a two-part process. The different percentiles of cholesterol all have similar slope variables but differing constants. Because they remain so static, we assume that people stay in the percentile rank for their entire life. The slope variables are regressed once based on the mean values, with a second-degree polynomial for men and a third-degree polynomial for women. A new constant was assigned for each percentile rank to predict the output line at which the absolute sum of the variance was minimized between the chart values and the predicted values using linear programming. In the model, a uniform distribution from 0 to 1 assigns patients a percentile rank. Their constant term is assigned at startup and then their actual cholesterol level is calculated each year based on their current age. HDL cholesterol does not vary with age, so only a single equation is used to assign each individual a lifetime HDL level. The percentile rank of total and HDL cholesterol are assigned independently.

## 4.4 Smoking Status

Smoking status is assigned randomly to each individual at model initiation. Smoking is assigned based on 2004 prevalence rates by race/ethnicity and sex (Maurice, Trosclair, and Merritt, 2005) (Table 4-1).

Table 4-1. Smoking Prevalence

| Race/Sex Group | Smoking Prevalence |
| --- | --- |
| White men | 0.241 |
| African American men | 0.239 |
| Hispanic men | 0.189 |
| White women | 0.204 |
| African American women | 0.172 |
| Hispanic women | 0.109 |

Source: Maurice, Trosclair, and Merritt (2005).

## 4.5 Left Ventricular Hypertrophy

LVH, like SBP, may be both a risk factor and a complication of CKD as it is associated with higher prevalence in CKD populations. Currently, LVH is assigned randomly at model initiation based on prevalence rates published in a review article by the Family Blood Pressure Program (16% for whites and 33% for African Americans; Hispanics are assumed to be 16%) (Table A‑7). Incident LVH is assigned only upon incidence of CKD based on a conditional probability of 0.6071 for whites and Hispanics and 0.5074 for African Americans as calculated from the weighted prevalence of LVH among CKD patients identified by Paoletti et al. (2005) and Cottone et al. (2007).

## 4.6 Obesity

Recent evidence suggests that rising obesity rates may be an important contributor to the increased prevalence of CKD (Hall et al., 2014). Obesity is a risk factor that leads to increased hypertension (Wilson et al. 2002; Garrison et al., 1987) and diabetes (Wilson et al. 2002; Ford et al., 1997; Resnick et al., 2000), which are both important risk factors for CKD (Hall et al., 2014). It is also thought theorized that obesity may have a direct effect on increasing the risk of CKD even after controlling for hypertension and diabetes although there is not strong evidence to support this (Hall et al., 2014; Elsayed et al. 2008; Stengel et al. 2003; de Boer et al. 2009; Foster et al. 2008).

Figure 4-1 depicts the how the relationship between obesity and CKD in the CKD model. We model the effect of obesity on increasing the risk for diabetes and hypertension. We allow for a direct effect of obesity on CKD, but set this to zero as default due to its uncertainty.

**Figure 4-1: Modeled Relationship between Obesity and CKD**

**Methods**

We conducted a systematic literature review to identify risk parameters for the effect of obesity on each factor. We used combinations of the following search terms for this literature review:

1. For the direct effect on CKD: “chronic renal insufficiency” OR “kidney failure, chronic” OR “chronic kidney disease” OR “chronic kidney failure”
2. For the effect on diabetes: “diabetes” OR “glucose tolerance” OR “HbA1c”
3. For the effect on hypertension: “hypertension” OR “blood pressure”

b. “obesity” OR “BMI”

c. “risk” OR “progression”

We also consulted with CDC team members to identify any additional articles that should be included in our review.

After identifying studies in our literature search, we selected the best studies for use in the model based on the following criteria:

1. Based on a longitudinal study
2. US-based population, preferably general population
3. Controls for appropriate variables (eg. diabetes and hypertension for the effect of obesity on CKD)

**Parameters**

We extracted parameters for the model from the studies we identified in our literature review. We extracted parameters for the effect of obesity on risk factors (diabetes and hypertension), CKD, and cardiovascular disease. We operationalized obesity into three categories: normal weight (BMI < 25), overweight (BMI 25 to 29.9), and obese (BMI 30+). We also extracted parameters for the change in BMI over time.

*The Effect of Obesity on Risk Factors*

Table 4-2 presents parameters for the effect of obesity on diabetes and hypertension. These parameters are drawn from Wilson et al. (2002) which uses data from the Framingham study.

**Table 4-**2: The Effect of Obesity on Risk Factors

| **Parameter** | **Value** | **Source** |
| --- | --- | --- |
| RR for Diabetes from |  | Wilson et al. 2002 |
| Overweight (BMI 25 to 29.9) | Men: 1.33  Women: 1 |
| Obese (BMI 30+) | Men: 2.12  Women: 1.42 |
| RR for Hypertension from |  | Wilson et al. 2002 |
| Overweight (BMI 25 to 29.9) | Men: 1.46  Women: 1.75 |
| Obese (BMI 30+) | Men: 2.21  Women: 2.75 |

*CKD*

We investigated evidence for a direct effect of obesity on CKD in addition to the indirect effect through diabetes and hypertension. We found that the majority of longitudinal studies that controlled for important factors such as diabetes and hypertension found no direct effect of obesity on CKD (Elsayed et al. 2008; Stengel et al. 2003; de Boer et al. 2009; Foster et al. 2008). Therefore, while we allow for a direct effect in the model, we set this relative risk parameter to 1 by default (Table 4-3)

**Table 4-**3: Effect of Obesity Directly on CKD

| **Parameter** | **Value** | **Source** |
| --- | --- | --- |
| RR for CKD from |  | Elsayed et al. 2008; Stengel et al. 2003; de Boer et al. 2009; Foster et al. 2008 |
| Overweight (BMI 25 to 29.9) | 1 |
| Obese (BMI 30+) | 1 |

*Cardiovascular Disease*

In order to account for competing risks in the model, we incorporate parameters for the effect of obesity directly on cardiovascular disease (CVD). Table 4-4 present parameters for the direct effect of obesity on CVD. We also considered including parameters for the effect of obesity on non-CVD mortality, but found that studies with a full set of controls estimated no effect on non-CVD mortality (Wilson et al. 2002).

**Table 4-**4: Effect of Obesity on Cardiovascular Disease

| **Parameter** | **Value** | **Source** |
| --- | --- | --- |
| RR for MI from |  | Wilson et al. 2002 |
| Overweight (BMI 25 to 29.9) | 1 |
| Obese (BMI 30+) | 1 |
| RR for CHD from |  |
| Overweight (BMI 25 to 29.9) | 1.43 |
| Obese (BMI 30+) | 1.58 |
| RR for Stroke from |  |
| Overweight (BMI 25 to 29.9) | 1 |
| Obese (BMI 30+) | 1 |

*Change over time*

Table 4-5 presents parameters for modeling changes in obesity over time. We use separate estimates for population between ages 30 and 49 and ages greater than 50.

**Table 4-5** Parameters for Obesity-CKD module

| **Parameter** | **Value** | **Source** |
| --- | --- | --- |
| Annual change in BMI for populations age 30 to 49 |  | Lewis et al. 2002 |
| White Male | 0.23 |
| White Female | 0.24 |
| Black Male | 0.32 |
| White Female | 0.41 |
| Annual change in BMI for populations age 50+ |  | Botoseneanu and Liag 2011 |
| White Male | 0.073 |
| White Female | 0.073 |
| Black Male | 0.020 |
| White Female | 0.020 |

# Complications

Complications are viewed as distinct from risk factors in the context of the chronic kidney disease (CKD) model as the incidence of complications is driven by progression of CKD and other risk factors. Three complications—coronary heart disease (CHD), stroke, and myocardial infarction (MI)—are assigned based on the Framingham risk equations (Anderson et al., 1990). The model does not use the Framingham risk equation for cardiovascular disease (CVD), instead assigning CVD to any agent who has CHD or stroke. CHD is assigned based on the Framingham risk equation, with a subset assigned to CHD with MI based on the Framingham risk equation for MI. Use of the Framingham equations entails tracking of certain covariates, including systolic blood pressure, total cholesterol, and high-density liproprotein (HDL) cholesterol. Left ventricular hypertrophy (LVH) is a risk factor considered in the Framingham equations but can be considered a complication because prevalence of LVH increases upon incidence of CKD.

As described in detail below, we use the Framingham risk equations, multiplied by a CKD risk factor, to determine the probability of CHD and stroke in the model. Neither the Framingham equations nor other CVD risk equations have been validated in a CKD population. A recent study by Weiner et al. (2006) reported that the Framingham instruments demonstrated poor overall accuracy in predicting cardiac events in individuals with CKD. Refit models (using the same explanatory variables but allowing for different parameter estimates) improved model accuracy; some of the key explanatory variables in the Framingham equation had less impact in the refit model. The study also found that the Framingham estimates generally underestimated the 5-year probability of cardiac events. Recalibration of the model (basically multiplying predicted probabilities by a factor greater than 1) improved prediction for women but not for men. The authors conjecture that the inability of recalibration to improve results may be driven by the competing risk of mortality, which was especially high (35% over 10 years) in men with CKD.

The Weiner et al. (2006) analysis suggests that our model could be improved if CVD equations that are validated for persons with CKD become available. In their absence, we will continue to use the Framingham equations because we also estimate CVD probabilities for individuals without CKD and with CKD stages 1 and 2 (most persons in the Weiner et al. study had CKD stage 3). Our multiplication by the CKD risk factor has the effect of recalibrating our CVD estimates upward to match the increased risk associated with CKD. In addition, our model includes the competing risk of death. If CVD equations that are validated for persons with CKD become available, we will be able to incorporate them into the model relatively simply.

## 5.1 Cardiovascular Disease

Assignment of CVD is based on patients having a stroke event or developing CHD, including by having an MI event. Patients who acquire CVD then face higher annual rates of stroke or myocardial events.

## 5.2 Coronary Heart Disease and Myocardial Infarction

Overall CHD is assigned based on probabilities derived from the Framingham CHD equation from Anderson et al. (1990) multiplied by a CKD stage multiplier. This probability is calculated annually considering sex, age, systolic blood pressure, cigarette use, total cholesterol, HDL cholesterol, diabetes, and LVH. For patients in CKD stages 3 or 4, this probability is then multiplied by a CKD stage hazard ratio of any CVD event based on data from Go et al. (2004) (Table 5-1). To help reduce output variance, the actual hazard ratio used in the model is based on a third-degree polynomial function fit to these data. CHD is assigned based on a randomly generated number resolving to less than the Framingham CHD probability * CKD stage hazard ratio.

Table 5-1. CKD Stage CVD Multipliers

| GFR | Hazard Ratio |
| --- | --- |
| 45–59 | 1.4 |
| 30–44 | 2.0 |
| 15–29 | 2.8 |

Source: Go et al. (2004).

Note: CKD = chronic kidney disease; CVD = cardiovascular disease; GFR = glomerular filtration rate

Patients who are assigned CHD in a given year are then allocated between MI and other CHD. The probability of MI is based on the Framingham MI equation multiplied by the CKD stage multipliers. The probability of non-MI CHD is assessed based on the differential between the Framingham equations for MI and CHD by using the same random number to assign both CHD and MI.

Patients face an increased probability of MI each year subsequent to being assigned CVD, either as CHD (including MI) or stroke. This probability is the same as above but then multiplied by a CVD multiplier of 2.19 based on the hazard rate of cardiac events among CVD patients taken from Weiner et al. (2006).

An MI event results in a 1-year increase in the probability of mortality. MI assigned in the first year of CVD is associated with mortality rates associated with a first MI event from Hunink et al. (1997). MI events incurred by patients with existing MI are assigned higher rates associated with a second MI (Table 6-3).

## 5.3 Stroke

Stroke is randomly assigned to patients annually based on the probability of a first stroke event and, after a first stroke, based on the probability of subsequent stroke events. The probability of an initial stroke event is based on the Framingham probability of stroke events. For CKD stages 3 and 4, this probability is multiplied by the CKD stage-specific hazard ratios for any CVD event.

Patients with CVD acquired due to either CHD (including MI) or stroke are subject to an increased probability of stroke in subsequent years based on the same probability function listed above multiplied by an existing CVD multiplier of 1.86 from Weiner et al. (2006) to reflect the increased risk of a stroke event among CVD patients.

# Mortality

During each period, persons in the normal stage and in chronic kidney disease (CKD) stages 1 through 4 can die from non-cardiovascular disease (CVD) causes and from CVD causes (as described below, mortality in stage 5 is handled differently, because United States Renal Data System [USRDS] data on death from end-stage renal disease [ESRD] provide a direct measure of mortality rates for this stage) (USRDS, 2008d). For each period, the annual probability of death, P(Death), is given by

P(Death) = P(nonCVD death) + Prob(CVD death).

We divide the overall probability into these two causes because the model separately generates the CVD complications of coronary heart disease (CHD) and stroke.

## 6.1 Non-CVD Deaths

To calculate P(non-CVD death), we start with the total mortality rate for each age, race/ethnicity, and sex group (Arias, 2006). We then subtract the CVD mortality rate for the corresponding group (NCHS worktable 210R, 2006). Finally, because Go et al. (2004) report that persons in CKD stages 3 and 4 have higher mortality rates than persons with glomerular filtration rate (GFR) > 60, we apply relative risk factors to individuals in this group. Thus,

P(nonCVD death) = P(all-cause death) − P(CVD death) if stage = normal, 1, 2
 = [P(all-cause death) − P(CVD death)] * RRstage if stage = 3, 4.

To estimate the RRstage, we manipulated Go et al.’s (2004) estimates. Adjusted hazard ratios estimated by Go et al. are presented in Table 6-1.

Table 6-1. Mortality Data Table from Go et al. (2004)

Source: Reproduced from Go et al. (2004).

Although these data do not provide hazard ratios for nonCVD causes, we can approximate this ratio by solving the following equation:

HRanycause = θ HRCVD + (1-θ) HRnonCVD

where θ, the share of CVD deaths, is 1/3. Solving this equation gives the following values:

| **Stage** | **GFR** | **HRnonCVD** |
| --- | --- | --- |
| Normal, 1, 2 | ≥ 60 | 1.0 |
| 3 | 45–59 | 1.1 |
| 3 | 30–44 | 1.7 |
| 4 | 15–29 | 3.4 |

Finally, interpreting HRnonCVD as an estimate of the relative risk, we get the estimates of P(nonCVD death) presented in Table 6-2.

Table 6-2. Relative Rates of CKD Mortality

| **Stage** | **GFR** | **P(nonCVD death)** |  | **RRnonCVD** |
| --- | --- | --- | --- | --- |
|  |  | P(nonCVD death) for persons with no CVD |  |  |
| Normal, 1, 2 | ≥ 60 | [P(all-cause death) − P(CVD death)] | X | 1.0 |
| 3 | 45–59 | [P(all-cause death) − P(CVD death)] | X | 1.1 |
| 3 | 30–44 | [P(all-cause death) − P(CVD death)] | X | 1.7 |
| 4 | 15–29 | [P(all-cause death) − P(CVD death)] | X | 3.4 |

The probability of death from all causes is based on 2003 census life tables adjusted to exclude mortality from heart disease and stroke, and the probability of CVD death comes from the National Center for Health Statistics (NCHS) (2006). Because life tables are only produced for whites and African Americans, Hispanics in the model are assigned mortality based on life tables for whites.

## 6.2 CVD Deaths

CVD deaths are related to the incidence of certain events in the model, including stroke, myocardial infarction (MI), and CKD. In any year in which a stroke occurs, the probability of death is increased by 0.142 for those under age 65 or by 0.321 for those over age 65 (Sacco et al., 1994). Similarly, higher rates of mortality are assigned in years in which a patient suffers an MI event. The excess mortality rate is based on age and whether the MI was the patients’ first or a subsequent MI, as shown in Table 6-3.

Table 6-3. Excess Mortality Due to Myocardial Infarction

| Age | First Myocardial Infarction | Subsequent Myocardial Infarction |
| --- | --- | --- |
| 30–44 | 0.015 | 0.087 |
| 45–54 | 0.034 | 0.112 |
| 55–64 | 0.073 | 0.145 |
| 65–74 | 0.159 | 0.187 |
| 75+ | 0.295 | 0.295 |

## 6.3 Stage 5 and ESRD Mortality

Patients in CKD stage 5 are often assumed to be captured in USRDS data under the ESRD benefit. However, in practice, it is likely that many patients who enter stage 5 do not immediately or ever initiate ESRD. We made a simplifying assumption that patients would not enter ESRD until their second year in stage 5. Thus, in the first year of stage 5 agents undergo the same mortality process as stages 3 and 4. For ESRD mortality, we identified mortality rates from the USRDS Renal Data Extraction and Referencing (RenDER) System available on the USRDS Web site (Table A‑8) (USRDS, 2008d). The data are from 2004 and include all ESRD modalities and all primary diagnoses and are restricted to the United States. Mortality rates are provided by age group, sex, race/ethnicity (white non-Hispanic, African American non-Hispanic, and Hispanic), and primary diagnosis (diabetes, hypertension, average of all other diagnoses). We created mortality rate look-up tables by fitting three-degree polynomial functions of age to each sex/race/risk factor group.

# Costs and Utility Values

The initial anticipated outcome measure for intervention evaluation is medical cost per quality-adjusted life year (QALY) gained. Therefore, the model tracks costs, utility, and life years incurred by simulated agents. However, the nature of costs and utility values will be highly dependent on the intervention under consideration, and thus the process of assigning model costs will continue to be modified and adapted for each use of the model. The natural history model described in this report includes three types of costs:

- annual expected medical costs for early stage chronic kidney disease (CKD) and complications costs,
- annual expected stage 5 and end-stage renal disease (ESRD) costs, and
- direct screening and treatment costs, which are discussed in Section 8-2.

The model produces effectiveness measures including life years, QALYs, and medical events and conditions. To incorporate time preferences, costs and QALYs are discounted 3% annually.

## 7.1 Early CKD Stage Costs

CKD stage costs are based on a cost function developed by Smith et al. (2007) that estimates costs for CKD and its related complications. This cost function is based on data from members of the Kaiser Permanente Northwest (KPNW) health maintenance organization (HMO), which has about 450,000 members who are representative of the Northwest area of the United States. The study estimated glomerular filtration rate (GFR), anemia, and the presence of proteinuria from available lab data; the presence of other comorbidities, such as coronary artery disease, congestive heart failure, hypertension, diabetes, hyperlipidemia, and peripheral vascular disease (PVD), was recorded according to ICD-9 codes in the claims.

The medical costs, defined as the amount actually paid by KPNW, include inpatient, outpatient, and pharmaceutical costs. Table 7-1 provides the model variables and their associated cost. To estimate costs for stages 1 and 2, a combination of the proteinuria and GFR parameters needs to be used. For parameters in the cost function that are not included in the model (i.e., anemia, congestive heart failure, hyperlipidemia, and PVD), we assumed a fixed cost value for each individual in the model equal to the prevalence of the condition by stage as reported by Smith et al. (2007) multiplied by the cost coefficient. All costs were inflated to 2006 dollars using the medical cost component of the Consumer Price Index (CPI).

Table 7-1. Annual Costs of CKD and Complications

| Covariate | Cost |
| --- | --- |
| Intercept | $1,666 |
| GFR 15–29 | $10,779 |
| GFR 30–59 | $5,781 |
| GFR 60–89 | $4,340 |
| Age | $36 |
| Men | −$208 |
| Proteinuria | $4,854 |
| Diabetes mellitus | $1,838 |
| Hypertension | $1,162 |
| Hyperlipidemia | 0 |
| Smoking | $474 |
| DM X GFR 60–89 | 0 |
| DM X GFR 30–59 | 0 |
| DM X GFR 15–29 | $4,031 |
| HTN X GFR 60–89 | −$1,373 |
| HTN X GFR 30–59 | −$2,046 |
| HTN X GFR 15–29 | −$3,065 |

Note: CKD = chronic kidney disease; DM = diabetes mellitus; GFR = glomerular filtration rate; HTN = hypertension.

## 7.2 ESRD Stage Costs

The Smith et al. (2007) cost function does not model costs associated with stage 5 CKD. In the model, we assume a 1‑year period between entering stage 5 CKD and progressing to ESRD. For the 1‑year period in stage 5 CKD prior to ESRD, we estimated costs as a combination of 6 months of stage 4 costs using estimates from Smith et al. ($7,902) and the costs for the 6 months just prior to the initiation of ESRD using costs from the United States Renal Data System (USRDS) ($13,409) (USRDS, 2006b, c, d). Thus, we estimated costs of $21,312 (2006 dollars) for the year of stage 5 CKD before the initiation of ESRD. For the ESRD costs, we used estimates from USRDS 2006 Annual Data Report (USRDS, 2006b, c, d). ESRD costs tend to spike in the months surrounding the initiation of dialysis and then level off in subsequent months. To capture this initial increase in costs, we calculated ESRD costs separately for the first year and subsequent years. USRDS reports total per capita ESRD costs of $57,841 (Table K4 in the 2006 Annual Data Report) and first month ESRD costs of $16,035 (2004 dollars). We inflated these estimates to 2006 dollars using the medical care component of the CPI and combined them with data from the USRDS Renal Data Extraction and Referencing (RenDER) data system on the total prevalent and incident populations to estimate monthly total costs.[[1]](#footnote-2) We estimated first year costs of $72,348 and subsequent year costs of $59,963 (2006 dollars) (USRDS, 2006b, c, d).

## 7.3 Effectiveness Measures

Although it is clearly important to ensure that the model can accurately measure the net cost of treatment and interventions, producing useful and comparable outcome measures is also vital. The primary effectiveness measure will be QALYs, which weighs life years lived by the agents’ health utility score in each year of life. Selected utility values by CKD stage are presented in Table 7-2. The primary benefit of a cost per QALY gained measure of effectiveness is that it can be compared with other, unrelated interventions. Disadvantages include the uncertainty associated with estimating health utility measures and the fact that utility may be less clinically relevant than specific medical outcomes, such as cases of ESRD avoided or life-years saved. The CKD model tracks both while medical outcomes will also be generated to produce more clinically relevant but less comparable results. Because the model tracks many specific medical events, the model can be used to track changes in incidence of medical events or person years lived with an intervention.

Table 7-2. Utility Values

| **Baseline** | **1** |
| --- | --- |
| **Annual Decrements** |  |
| Proteinuria | 0.01 |
| GFR 30–59 | 0.05 |
| GFR 15–29 | 0.07 |
| GFR <15 | 0.20 |
| Stroke, ever | 0.582 |
| CA/MI, current year | 0.12 |
| CHD, ever without MI | 0.053 |

Note: CA = cardiac arrest; CHD = coronary heart disease; GFR = glomerular filtration rate; MI = myocardial infarction.

We obtained quality of life values, in the form of QALYs, for the different stages of CKD primarily from Gorodetskaya et al. (2005). For GFR values, we used adjusted values of the QALYs estimated by Gorodetskaya et al., who used a time trade-off method. An adjustment was necessary because the QALYs for the highest GFR range (≥ 60) estimated by Gorodetskaya et al. were less than 1. Because an individual with a GFR ≥ 60 and no proteinuria represented an individual with no CKD, we wanted to set this as the baseline QALY level. We thus adjusted each of the QALY values for the subsequent GFR levels to be the difference relative to the baseline of GFR ≥ 60 and no proteinuria. Gorodetskaya et al. also made no distinction between having proteinuria and not having proteinuria, so we included a QALY decrement of 0.01 used by Boulware et al. (2003).

We also included QALY values for the complications related to CKD, which we obtained from several sources in the literature (also see Table 7-2). We used a value of 0.88 for an individual who had a myocardial infarction (MI) and survived (Tsevat et al., 1993) and a value of 0.947 for angina, which is a weighted average of two severity groups from Nease et al. (1995). For stroke, we used a value of 0.418, which was a weighted average of QALYs for minor, moderate, and severe strokes from Meenan et al. (2007).

# Medical Care and Interventions

## 8.1 Integration of Hypothetical Treatment Scenarios

The focus of the model development was to develop a complete and accurate simulation of the incidence and progression of early-stage chronic kidney disease (CKD) to facilitate the integration of a wide array of possible interventions. Table 8-1 lists model parameters, their baseline values, and the source of the value.

The primary analysis simulates screening for micro- and macroalbuminuria followed by treatment with angiotensin-converting enzyme (ACE) inhibitors or angiotensin II receptor blockers (ARBs). As Figure 8-1 shows, each person who undergoes screening receives an initial quantitative microalbuminuria screening test. If the first test returns positive, a second confirmatory test is administered as well as tests to estimate glomerular filtration rate (GFR). Once microalbuminuria has been confirmed, persons with GFR less than 60 receive additional tests to determine the cause of CKD and then receive ACE or ARB therapy. On the other hand, persons with GFR greater than or equal to 60 proceed directly from the confirmation of microalbuminuria to ACE or ARB therapy.

Interventions are defined by the rate of screening employed. We included a “No Screening” scenario in which persons undergo no screening and no treatment until end-stage renal disease (ESRD), at which time costs and mortality are assumed to equal observed USRDS data. We then introduced universal screening based on annual, 2-year, 5-year, or 10-year intervals beginning at age 50.

Although the “No Screening” scenario is useful for comparing the impact of an intervention in a previously untreated population, it may overstate the benefit of an intervention in a population that already undergoes some screening and treatment as part of usual care. We employ a “Usual Care” scenario that introduces expected annual probabilities of microalbuminuria screening: 23% for patients with diabetes and hypertension, 22% for patients with diabetes only, 2% for patients with hypertension only, and 0% for patients with neither diabetes nor hypertension (USRDS, 2006e).

Screening for microalbuminuria is associated with a sensitivity of 76% and specificity of 96% (Sarafidis et al., 2008). To account for lower than ideal adherence, treatment was assumed to initiate following diagnosis in 75% of persons (Boulware et al., 2003). The effect of ACE/ARB therapy on persons with microalbuminuria is assumed to be a 55% reduction in the probability of progression from micro- to macroalbuminuria (Strippoli et al., 2004). For those with macroalbuminuria, treatment is assumed to provide a 23% reduction in annual mortality rates and—based on an analysis of the literature—a 32.7% reduction in annual GFR decrement (see Table 8-2) (Agodoa et al., 2001; Ruggenenti et al., 1997, 1999).

Table 8-1. Selected Model Parameters

| **Variable** | **Value** | | **Source** |
| --- | --- | --- | --- |
| Discount Rate | 0.03 | | Assumption |
| **Population** |  | |  |
| Initial age | 30 | | Assumption |
| Age 30 prevalence by race/ethnicity, sex | Report Table A.1 | | 2005 U.S. Census estimate |
| **Natural History** |  | |  |
| Age 30 initial GFR |  | |  |
| Mean | 101.941 | | NHANES |
| Standard deviation (normal) | 19.313 | | NHANES |
| Relative GFR decline observed in NHANES | 0.65332 | | NHANES |
| **Mortality** |  | |  |
| Annual background mortality, function of age, race/ethnicity, sex | Report Table A.13 | | 2003 Census Life Tables (Arias, 2006) |
| ESRD mortality, varies by age, diabetes, hypertension | Report Table A.9 | | USRDS (2008e) |
| Mortality coefficient, function of GFR | Report, § 6.1 | | Go et al. (2004), assumption |
| Microalbuminuria incidence | Report Table A.2 | | NHANES, Adler et al. (2003), Mann et al. (2003) |
| Macroalbuminuria transitions | Report Table A.3 | | NHANES, Adler et al. (2003) |
| Excess mortality due to stroke |  | | Sacco et al. (1994) |
| Age < 65 | 0.142 | |  |
| Age ≥ 65 | 0.321 | |  |
| Excess mortality due to MI, by age | **First MI** | **Other MI** | Hunink et al. (1997), Weinstein et al. (1987) |
| 30–44 | 0.0154 | 0.0867 |
| 45–54 | 0.0336 | 0.112 |  |
| 55–64 | 0.073 | 0.1446 |  |
| 65–74 | 0.1587 | 0.1867 |  |
| 75+ | 0.2953 | 0.2953 |  |
| **Risk Factors and Complications** |  | |  |
| HDL level, varies by sex, constant with age | Report Function A-6 | | NHANES data |
| Total cholesterol level, varies by sex and age | Report Function A-6 | | NHANES data |
| Systolic blood pressure, varies by age, sex, race/ethnicity, CKD | Report Function A-5 | | NHANES data |
| Diabetes incidence, varies by age, race/ethnicity, sex | Report Table A‑4 | | Cowie et al. (2006), Geiss et al. (2006) |
| Prevalence of smoking | Report Table 4-1 | | Maurice et al. (2005) |
| LVH, prevalence and incidence with CKD | Report Table A‑8 | | Family Blood Pressure Program, Paoletti et al. (2005), Cottone et al. (2007) |

(continued)

Table 8-1. Selected Model Parameters (continued)

| **Variable** | **Value** | **Source** |
| --- | --- | --- |
| **Risk Factors and Complications (continued)** |  |  |
| Hazard ratio for cardiac events for existing CVD patients | 2.19 | Weiner et al. (2006) |
| Hazard rate for stroke for existing CVD patients | 1.86 | Weiner et al. (2006) |
| CVD event coefficient, function of GFR | Report Function 5-1 | Go et al. (2004) |
| Framingham CHD probability | Report, § 5.2 | Anderson et al. (1990) |
| Framingham MI probability | Report, § 5.2 | Anderson et al. (1990) |
| Framingham stroke probability | Report, § 5.3 | Anderson et al. (1990) |
| **Utility Rates** |  |  |
| Background Utility | 1 | Assumption |
| Macroalbuminuria coefficient | 0.01 | Boulware et al. (2003) |
| GFR 30–59 coefficient | 0.05 | Gorodetskaya et al. (2005) |
| GFR 15–29 coefficient | 0.07 | Gorodetskaya et al. (2005) |
| GFR < 15 coefficient | 0.2 | Gorodetskaya et al. (2005) |
| Stroke, ever coefficient | 0.582 | Gorodetskaya et al. (2005) |
| MI coefficient | 0.12 | Gorodetskaya et al. (2005) |
| Non MI CHD coefficient | 0.053 | Gorodetskaya et al. (2005) |
| **Intervention Parameters** |  |  |
| Sensitivity of testing for microalbuminuria | 0.73 | Sarafidis et al. (2008) |
| Specificity of testing for microalbuminuria | 0.96 | Sarafidis et al. (2008) |
| Probability of biopsy during screening |  |  |
| Age <65, macroalbuminuria, no DM, no HT | 0.90 | Personal communication with Neil Powe (2008) |
| Age <65, no macroalbuminuria, no DM, no HT | 0.40 | Personal communication with Neil Powe (2008) |
| Age 65+, no DM, no HT | 0.25 | Personal communication with Neil Powe (2008) |
| DM or HT | 0.05 | Boulware et al. (2003) |
| Treatment adherence | 0.75 | Boulware et al. (2003) |
| Treatment Coefficients |  |  |
| Micro to macroalbuminuria transition | 0.45 | Strippoli et al. 2004 |
| Mortality | 0.77 | Boulware et al. (2003) |
| Annual GFR | 0.673 | Agodoa et al. (2001), Ruggenenti et al. (1997, 1999) |

Figure 8-1. Schematic of Screen and Treat Intervention

Table 8-2. Literature Review of Effect of ACE Inhibitor Use on GFR Progression

| Study | Reduction in Rate of GFR Decline |
| --- | --- |
| Agodoa et al. (2001) | 35.9% |
| Ruggenenti et al. (1997) | 39.8% |
| Ruggenenti et al. (1999) | 22.5% |

Our primary scenarios involved universal screening for U.S. adults beginning at age 50. We duplicated this analysis in subpopulations with diabetes, hypertension but no diabetes, and neither hypertension nor diabetes. We also investigated the impact of starting universal screening at alternative ages.

## 8.2 Screening and Treatment Costs

Simulating medical interventions will necessitate tracking certain event-specific costs, such as costs associated with screening and treatment. Only costs that are directly impacted by an intervention need to be considered, and thus the costs included will be dependent on the intervention under consideration. For the example intervention described in this report, direct costs include costs for microalbuminuria screening, any recommended specialist or diagnostic testing, and the cost of ongoing medical care and pharmaceutical treatment. The costs are identical for both the control and intervention groups, as the intervention only seeks to increase the rate of background screening.

The costs of screening include an initial physician visit to measure urine albumin and creatinine levels in order to identify the presence of microalbuminuria. If the microalbuminuria test is positive, then the screening costs also include a second physician visit to confirm the presence of microalbuminuria. The second visit also includes a measurement of serum creatinine to estimate GFR. The costs for the physician visit and diagnostic test are both estimated using Medicare reimbursement rates (CMS physician fee schedule, 2007; CMS clinical lab fee schedule, 2007). See Table 8-3 for each cost.

Once the presence of microalbuminuria has been confirmed, persons receive additional diagnostic costs if their GFR is less than 60 ml/min per 1.73 m^2^. The tests included in the one-time diagnosis costs are those listed in Boulware et al. (2006). To calculate the cost associated with each test, we multiplied the probability of the test being recommended (Boulware et al., 2006) by the Medicare reimbursement rate (CMS clinical lab fee schedule, 2007). Additionally, each person has a probability of receiving a renal biopsy depending on age, the level of albuminuria, diabetes, and hypertension. Rates of renal biopsy and probability of complications were based on Boulware et al. (2003) and personal communication with one of the authors (personal communication, Neil Powe, 2008). The cost for each renal biopsy was based on Medicare reimbursement rates (CMS physician fee schedule, 2007; CMS clinical lab fee schedule, 2007) and Healthcare Cost and Utilization Project (HCUP) data for the hospital costs (AHRQ, 2005). See Table 8-3 for each cost.

Persons with microalbuminuria also receive annual treatment costs. Depending on the presence of diabetes and hypertension, each person with microalbuminuria received either angiotensin-converting enzyme (ACE) inhibitors (hypertension or neither) or angiotensin II receptor blockers (ARBs) (diabetes). The dosage taken for each of the drugs was based on Rodby et al. (2003), Lewis et al. (2001), Nakao et al. (2003), and Jafar et al. (2001), while the unit costs were based on prices listed in the Redbook (Drug Topics RedBook, 2007). Using rates from Boulware et al. (2003), additional complication costs related to the use of ACE inhibitors or ARBs were also included. Costs associated with the complications were estimated using Medicare reimbursement rates and the mean emergency department costs from each state included in the State Emergency Department Databases (SEDD) (2004). All persons with neither diabetes nor hypertension receive three additional annual generalist visits, based on an assumption by Boulware et al. (2003). Persons with a GFR < 60 receive an annual visit to monitor the progress of CKD. We used the tests listed by Boulware et al. (2003) for the annual specialist visit and combined these with Medicare reimbursement rates (CMS physician fee schedule, 2007; CMS clinical lab fee schedule, 2007). The costs were slightly higher for the diabetes population than for the hypertension or neither hypertension nor diabetes populations. See Table 8-3 for each cost.

Table 8-3. Aggregated Intervention Costs

| Intervention Component | Cost |
| --- | --- |
| Screening |  |
| Initial visit | $80.32 |
| Second visit if positive during first visit | $62.64 |
| One time diagnosis costs if GFR <60 |  |
| Diabetes or hypertension | $346.74 |
| Neither hypertension nor diabetes |  |
| Macroalbuminuria and age < 65 | $2,590.57 |
| Microalbuminuria and age < 65 | $1,270.67 |
| Age ≥65 | $874.70 |
| Annual specialist follow-up if GFR <60 |  |
| Diabetes | $85.37 |
| Hypertension or neither | $77.28 |
| Annual generalist visits (3) |  |
| Neither | $120.48 |
| Drug therapy |  |
| Diabetes | $478.28 |
| Hypertension or neither | $190.44 |

# Race-specific Progression Calibration

The model includes certain race-specific risk factors that results in predicting higher chronic kidney disease (CKD) prevalence and end-stage renal disease (ESRD) incidence among African Americans than other races. However, the baseline model results for African Americans may not accurately reflect CKD progression rates. In National Health and Nutrition Examination Survey (NHANES) data, the prevalence of CKD among African Americans is 0.147, whereas United States Renal Data System (USRDS) data indicate a lifetime incidence rate of ESRD of 0.0863. The original model yields CKD prevalence of 0.143 and ESRD incidence of 0.0451 for African Americans. While the model reasonably predicts CKD prevalence, it far underestimates ESRD rates among African Americans. Conversely, the baseline model slightly underpredicts CKD prevalence but overpredicts ESRD incidence among whites. Thus, it appears that the model underestimates the rate of progression to ESRD among African Americans.

In this section, we describe risk factors that increase CKD progression rates among African Americans. We show that these risk factors lead to faster progression to ESRD among African Americans, but that the impact of these risk factors is not sufficient to account for the wide race disparity in ESRD rates observed in USRDS data. We then test the impact of other potential factors that could further increase CKD progression in African Americans but were not included in the model. Finally, we show the GFR calibration process by which we increased CKD progression among African Americans with stage 3 or worse CKD to match ESRD incidence rates. We show the impact of each risk factor on validation outcomes by incorporating African American-specific risk levels in a version of the model in which African Americans otherwise face no additional risk factors except background mortality.

Briefly, our results show that three primary existing African American risk factors included in the model (elevated systolic blood pressure, higher diabetes incidence, and higher micro- and macroalbuminuria) each speed progression of CKD and in turn increase incidence of ESRD among African Americans. However, the impact of these risk factors is not nearly enough to fully explain the much higher rates of ESRD identified among African Americans in USRDS data. Therefore, in addition to fully accounting for African American risk factors, we calibrated the model using glomerular filtration rate (GFR) reduction coefficients to speed progression of African Americans in stages 3 and 4 in order to replicate the ESRD rates observed in the African American population.

## 9.1 African American CKD Progression Risk Factors

### 9.1.1 Blood Pressure

The model accurately recreates the distribution of systolic blood pressure (SBP) values in NHANES by race/ethnicity, sex, and age. SBP above 140 indicates hypertension (HTN). HTN results in faster GFR reduction and higher incidence of micro- and macroalbuminuria, which in turn further accelerate GFR decline. The rates of GFR decline from SBP are taken from Boulware et al. (2006).

The impact of race-specific blood pressure distributions on the progression of CKD among African Americans is shown in Table 9‑1. Higher SBP values for African Americans and lower values for non-African Americans improve model agreement with external validation targets for both African Americans and non-African Americans; however the effect is very small, increasing ESRD incidence by only 0.001. Despite the small impact of race-specific blood pressure, we retain race-specific blood pressure values as these are supported by NHANES data.

Table 9-1. Impact of Race-Specific Blood Pressure Values on CKD Progression among African Americans

| Scenario | ESRD Incidence | Age-Adjusted Prevalence | | | | | |
| --- | --- | --- | --- | --- | --- | --- | --- |
|  |  | Any CKD | Stage 1 | Stage 2 | Stage 3 | Stage 4 | Stage 5 |
| Baseline^a^ | 0.039 | 0.143 | 0.027 | 0.035 | 0.072 | 0.007 | 0.002 |
| African American—specific SBP | 0.039 | 0.144 | 0.027 | 0.036 | 0.073 | 0.007 | 0.002 |

^a^Baseline results based on no race-specific risk factors or parameters except mortality rates.

### 9.1.2 Diabetes Prevalence

Like SBP, the model accurately represents incidence of diabetes by sex, race, and age. Diabetes results in larger annual declines in GFR based on Boulware et al. multipliers. Higher prevalence of diabetes in African Americans results in faster progression overall for African Americans and increases ESRD incidence from 0.039 to 0.047. Increasing the Boulware et al.-derived GFR decline multiplier would increase progression for African Americans and non-African Americans. Thus, while this change would improve external validation performance of the model for African Americans, it leads to a greater overestimates of ESRD incidence among non-African Americans. The impact of race-specific diabetes incidence and prevalence rates on CKD progression among African Americans is shown in Table 9‑2.

Table 9-2. Impact of Race-Specific Diabetes Prevalence and Incidence on CKD Progression among African Americans

| Scenario | ESRD Incidence | Age-Adjusted Prevalence | | | | | |
| --- | --- | --- | --- | --- | --- | --- | --- |
|  |  | Any CKD | Stage 1 | Stage 2 | Stage 3 | Stage 4 | Stage 5 |
| Baseline^a^ | 0.039 | 0.143 | 0.027 | 0.035 | 0.072 | 0.007 | 0.002 |
| African American—specific DM prevalence | 0.048 | 0.148 | 0.027 | 0.036 | 0.075 | 0.008 | 0.003 |

^a^Baseline results based on no race-specific risk factors or parameters except mortality rates.

### 9.1.3 Albuminuria Prevalence

Albuminuria is an indication for stage 1 and 2 CKD and contributes to faster GFR decline. The model differentiates between micro- and macroalbuminuria. Macroalbuminuria results in patients experiencing larger annual GFR decrements based on values derived by Boulware et al. 2006. In the baseline model, we analyzed the prevalence of albuminuria in NHANES data by HTN, diabetes, sex, and age and fit prevalence curves. We then solved for microalbuminuria incidence rates that would resolve to these prevalence rates. We then solved for micro to macro transition rates to reflect the prevalence of each respective condition. For the current analysis, we updated the original process in two ways: (1) we added race as one of the differentiated factors, and (2) we refined the original approach by using an iterative process to solve for the micro incidence and macro transition rates such that model output matches NHANES prevalence data. Because albuminuria is associated with higher mortality in the model, without this second step the model was underpredicting albuminuria prevalence at older ages, especially for African Americans.

African Americans have higher prevalence of albuminuria, especially macroalbuminuria. GFR reduction multipliers for macroalbuminuria based on those derived by Boulware et al. 2006 lead to much faster CKD progression in the model. Thus, incorporating racial differences for albuminuria increases CKD progression among African Americans as shown in Table 9‑3. However, the relative impact is still far too small to account for the ESRD disparity shown in the validation data. One consideration that may be investigated is the impact of different actual values of albuminuria. Macroalbuminuria is defined as albumin/creatinine ratio (ACR) > 300 µg albumin/mg creatinine. In NHANES data, the average ACR among whites with macroalbuminuria is 1,023 µg albumin/mg creatinine, compared with 1,514 among African Americans. We do not currently account for differences in progression based on severity of macroalbuminuria due to a lack of data.

Table 9-3. Impact of Race-Specific Microalbuminuria Incidence and Transition to Macroalbuminuria on CKD Progression among African Americans

| Scenario | ESRD Incidence | Age-Adjusted Prevalence | | | | | |
| --- | --- | --- | --- | --- | --- | --- | --- |
|  |  | Any CKD | Stage 1 | Stage 2 | Stage 3 | Stage 4 | Stage 5 |
| Baseline^a^ | 0.039 | 0.143 | 0.027 | 0.035 | 0.072 | 0.007 | 0.002 |
| African American—specific albuminuria prevalence | 0.042 | 0.181 | 0.046 | 0.052 | 0.073 | 0.007 | 0.003 |

^a^Baseline results based on no race-specific risk factors or parameters except mortality rates.

## 9.2 Other Potential Factors in CKD Progression among African Americans

### 9.2.1 Lower Medical Care Rates

We ran our validation exercises based on the assumption of usual standards of care. These include background screening rates followed by recommended standards of care. The background screening rates are from data reported by the USRDS and do not vary by race. It is possible that African Americans have lower screening rates or lower treatment rates following diagnosis. This would presumably result in higher rates of ESRD incidence. However, when we assume an extreme of zero treatment among African Americans, their ESRD incidence only slightly increased (Table 9-4). Because of this and a lack of supporting data, we did not alter the medical care rates for African Americans.

Table 9-4. Impact of No Preventive Medical Care on CKD Progression among African Americans

| Scenario | ESRD Incidence | Age-Adjusted Prevalence | | | | | |
| --- | --- | --- | --- | --- | --- | --- | --- |
|  |  | Any CKD | Stage 1 | Stage 2 | Stage 3 | Stage 4 | Stage 5 |
| Baseline^a^ | 0.039 | 0.143 | 0.027 | 0.035 | 0.072 | 0.007 | 0.002 |
| No Preventive Care | 0.040 | 0.143 | 0.027 | 0.035 | 0.073 | 0.007 | 0.002 |

^a^Baseline results based on no race-specific risk factors or parameters except mortality rates.

### 9.2.2 ESRD Entry Immediately Upon Entering Stage 5

In the model, ESRD begins after survival of 1 year in stage 5. We lack data on rates of ESRD entry by stage and stage duration. This 1-year delay is based on an assumption that not all persons in stage 5 would enter ESRD immediately, and USRDS data found that on average, there was approximately an 11-month delay before ESRDS was initiated. We tested the impact that all persons who enter stage 5 would immediately begin ESRD. We find the ESRD incidence increases from 0.039 to 0.045, indicating that it is possible that our baseline assumption is overly conservative (Table 9-5). However, applying this assumption

Table 9-5. Impact of Immediate Entry to ESRD upon Initiation of Stage 5 on CKD Progression among African Americans

| Scenario | ESRD Incidence | Age-Adjusted Prevalence | | | | | |
| --- | --- | --- | --- | --- | --- | --- | --- |
|  |  | Any CKD | Stage 1 | Stage 2 | Stage 3 | Stage 4 | Stage 5 |
| Baseline^a^ | 0.039 | 0.143 | 0.027 | 0.035 | 0.072 | 0.007 | 0.002 |
| No ESRD entry delay | 0.045 | 0.143 | 0.027 | 0.035 | 0.073 | 0.007 | 0.002 |

^a^Baseline results based on no race-specific risk factors or parameters except mortality rates.

to non-African Americans would exacerbate the already overly high ESRD incidence rate found over the entire population, and it may not be plausible that only African Americans do not experience a delay in ESRD entry. Thus, we retain the ESRD entry delay of 1 year after initiation of stage 5.

### 9.2.3 Race-specific GFR Initial Distribution and Annual Decline

In the model, GFR is assigned an initial (age 30) distribution based on the distribution of GFR values identified among 25- to 35-year-olds in NHANES (mean=101.9409, SD=19.313). GFR is then reduced annually based on an annual decrement value multiplied by certain risk factor coefficients. The decrement is based on the mean annualized difference in mean GFR values between 30- and 80-year-olds observed in NHANES data (0.65332). The annual decline value is then multiplied by coefficients based on macroalbuminuria, diabetes, and hypertension.

The MDRD equation includes a 1.21 coefficient for African Americans, which is factored into the overall GFR values calculated from NHANES data. In the original model, African Americans are assigned the same GFR parameters as the rest of the population. We considered the impact of calculating GFR parameters separately by race. Doing this, we find African Americans have higher initial GFR values (111.575) and a higher annual decrement (0.757). This increases ESRD incidence by 0.001 with a similar increase in CKD prevalence (Table 9-6). Due to the small impact of this change, we elected to retain the non-race specific GFR distributions and rely on the race coefficient in the MDRD equation to account for racial differences.

Table 9-6. Impact of Race-Specific GFR Distributions on CKD Progression among African Americans

| Scenario | ESRD Incidence | Age-Adjusted Prevalence | | | | | |
| --- | --- | --- | --- | --- | --- | --- | --- |
|  |  | Any CKD | Stage 1 | Stage 2 | Stage 3 | Stage 4 | Stage 5 |
| Baseline^a^ | 0.039 | 0.143 | 0.027 | 0.035 | 0.072 | 0.007 | 0.002 |
| African American—specific GFR distribution | 0.040 | 0.144 | 0.040 | 0.026 | 0.067 | 0.008 | 0.003 |

^a^Baseline results based on no race-specific risk factors or parameters except mortality rates.

## 9.3 Calibration of GFR to Match African American ESRD Incidence Rates

Even though the model includes higher albuminuria rates, diabetes prevalence, and blood pressure for African Americans than for other groups, it continues to underpredict ESRD rates for African Americans and overstate ESRD for others. We considered several options to alter the model’s GFR progression process to account for racial differences in progression.

Because of the overprediction of CKD using race-specific GFR values, we considered other options for calibrating the universal GFR values based on race. Based on the albuminuria calibration results, overall CKD prevalence closely matches NHANES data, but the prevalence of stage 3 is too high, whereas the prevalence of stages 4 and 5 are too low. We applied a multiplier to the GFR decrement value when African Americans reached stage 3 (3x) and again at stage 4 (6x). Based on these calibration settings, overall CKD prevalence and the prevalence of each individual stage closely match prevalence values found in NHANES data. In this scenario, the incidence of ESRD reaches 7.8%, less than 1 percentage point less than the 8.6% identified in USRDS data (Table 9‑7).

Table 9-7. Impact of Race-Specific GFR Multipliers on CKD Progression among African Americans

| Scenario | ESRD Incidence | Age-Adjusted Prevalence | | | | | |
| --- | --- | --- | --- | --- | --- | --- | --- |
|  |  | Any CKD | Stage 1 | Stage 2 | Stage 3 | Stage 4 | Stage 5 |
| Base model^a^ | 0.039 | 0.143 | 0.027 | 0.035 | 0.072 | 0.007 | 0.002 |
| African American risk factors | 0.052 | 0.182 | 0.048 | 0.051 | 0.072 | 0.008 | 0.003 |
| African American risk factors and GFR calibration | 0.082 | 0.181 | 0.046 | 0.053 | 0.067 | 0.009 | 0.005 |
| Observed outcomes^b^ | 0.086 | 0.147 | 0.033 | 0.040 | 0.062 | 0.005 | — |

^a^Baseline results based on no race-specific risk factors or parameters except mortality rates.

^b^Observed stage-specific CKD prevalence is based on the National Health and Nutrition Examination Survey (NHANES), 1999–2006. We did not report the estimated prevalence of stage 5 due to the limited sample size in NHANES. ESRD lifetime incidence is based on data from the United States Renal Data System.

# Model Validation

## 10.1 Validation Process

We validated the model according to recommended standards outlined by the International Society for Pharmacoeconomics and Outcomes Research (ISPOR) Task Force (Weinstein et al., 2003). Model testing and validation continue throughout model construction. Each component of the model must undergo testing followed by internal and external validation. Testing ensures that there are no programming errors in the model. Internal validation tests the data and logic of the model to ensure that the model is correctly generating the results. External validation, when possible, provides a sense of confidence in the model in that it agrees with data sources that were not used in the development of the model. All subsequent validation results are based on the Usual Care scenario.

## 10.2 Parameterization Testing and Internal Validation

Model testing and internal validation start by assessing the proper parameterization of the model. This includes model processes that are used to distribute demographic characteristics and risk factor parameters across the population, such as race/ethnicity, sex, systolic blood pressure (SBP), total and high-density lipoprotein (HDL) cholesterol, smoking status, and left ventricular hypertrophy (LVH). Tables 10-1, 10-2, and 10-3 demonstrate internal validity of model processes to distribute SBP and total and HDL cholesterol across the population based on the National Health and Nutrition Examination Survey (NHANES) data. Binary variables such as smoking and LVH prevalence are tested based on simple prevalence.

Table 10-1. Internal Validation Results, SBP in Non-CKD Men

| **Source** | **Age** | **Percentile Rank** | | | | | | | | | | |
| --- | --- | --- | --- | --- | --- | --- | --- | --- | --- | --- | --- | --- |
|  |  | **0%** | **5%** | **10%** | **15%** | **25%** | **50%** | **75%** | **85%** | **90%** | **95%** | **100%** |
| Model | 30 | 98 | 102 | 104 | 107 | 110 | 115 | 123 | 128 | 132 | 136 | 142 |
| NHANES | 37 | — | 104 | 107 | 109 | 112 | 119 | 126 | 130 | 134 | 140 | — |
| Model | 37 | 100 | 103 | 106 | 109 | 112 | 118 | 127 | 133 | 137 | 141 | 147 |
| NHANES | 49.5 | — | 105 | 109 | 111 | 115 | 124 | 134 | 140 | 145 | 153 | — |
| Model | 49.5 | 102 | 106 | 109 | 112 | 117 | 124 | 134 | 141 | 145 | 150 | 156 |
| NHANES | 59.5 | — | 107 | 111 | 114 | 119 | 128 | 141 | 148 | 152 | 159 | — |
| Model | 59.5 | 103 | 108 | 112 | 115 | 120 | 129 | 139 | 147 | 152 | 157 | 164 |
| NHANES | 69.5 | — | 111 | 116 | 120 | 125 | 136 | 147 | 156 | 160 | 168 | — |
| Model | 69.5 | 105 | 110 | 114 | 118 | 124 | 133 | 145 | 153 | 158 | 164 | 171 |
| NHANES | 79.5 | — | 112 | 118 | 122 | 127 | 138 | 150 | 158 | 162 | 168 | — |
| Model | 79.5 | 107 | 112 | 117 | 121 | 127 | 138 | 151 | 160 | 165 | 171 | 179 |
| NHANES | 90 | — | 115 | 119 | 123 | 129 | 143 | 157 | 164 | 170 | 181 | — |
| Model | 90 | 109 | 115 | 120 | 124 | 131 | 143 | 157 | 166 | 172 | 179 | 187 |
| Model | 100 | 110 | 117 | 122 | 127 | 134 | 147 | 163 | 173 | 179 | 186 | 194 |

Table 10-2. Internal Validation Results, Total Cholesterol in Men

| **Source** | **Age** | **Percentile Rank** | | | | | | | | |
| --- | --- | --- | --- | --- | --- | --- | --- | --- | --- | --- |
|  |  | **5%** | **10%** | **15%** | **25%** | **50%** | **75%** | **85%** | **90%** | **95%** |
| NHANES | 20–34 | 131 | 142 | 148 | 161 | 183 | 209 | 223 | 233 | 253 |
| Model | 27 | 127 | 138 | 147 | 161 | 185 | 212 | 230 | 241 | 254 |
| NHANES | 35-44 | 143 | 154 | 163 | 180 | 205 | 232 | 247 | 257 | 267 |
| Model | 39.5 | 145 | 155 | 164 | 179 | 203 | 230 | 247 | 259 | 271 |
| NHANES | 45-54 | 154 | 167 | 178 | 191 | 214 | 242 | 255 | 266 | 283 |
| Model | 49.5 | 153 | 164 | 173 | 187 | 211 | 238 | 256 | 267 | 280 |
| NHANES | 55–64 | 154 | 167 | 174 | 189 | 214 | 243 | 258 | 270 | 282 |
| Model | 59.5 | 157 | 167 | 176 | 191 | 215 | 242 | 259 | 271 | 283 |
| NHANES | 65–74 | 149 | 163 | 175 | 186 | 209 | 237 | 248 | 263 | 284 |
| Model | 69.5 | 155 | 166 | 175 | 189 | 213 | 240 | 258 | 269 | 282 |
| NHANES | 75+ | 145 | 155 | 164 | 176 | 203 | 230 | 246 | 255 | 273 |
| Model | 85 | 143 | 154 | 163 | 177 | 201 | 228 | 246 | 257 | 270 |

Table 10-3. Internal Validation Results, HDL Cholesterol

| **Source** | **Age** | **Percentile Rank** | | | | | | | | |
| --- | --- | --- | --- | --- | --- | --- | --- | --- | --- | --- |
|  |  | **5%** | **10%** | **15%** | **25%** | **50%** | **75%** | **85%** | **90%** | **95%** |
| **Men** |  |  |  |  |  |  |  |  |  |  |
| Model | All | 28 | 31 | 33 | 37 | 44 | 53 | 58 | 62 | 66 |
| NHANES | 20–34 | 28 | 32 | 34 | 38 | 45 | 53 | 59 | 62 | 69 |
|  | 35–44 | 28 | 30 | 32 | 36 | 43 | 52 | 57 | 61 | 73 |
|  | 45–54 | 26 | 30 | 32 | 35 | 42 | 52 | 58 | 66 | 75 |
|  | 55–64 | 28 | 31 | 34 | 36 | 42 | 51 | 57 | 61 | 71 |
|  | 65–74 | 28 | 30 | 32 | 36 | 43 | 54 | 58 | 64 | 73 |
|  | 75+ | 28 | 31 | 34 | 37 | 44 | 54 | 61 | 66 | 75 |
| **Women** |  |  |  |  |  |  |  |  |  |  |
| Model | All | 34 | 38 | 40 | 45 | 53 | 64 | 71 | 75 | 80 |
| NHANES | 20–34 | 34 | 38 | 41 | 45 | 53 | 64 | 69 | 74 | 83 |
|  | 35–44 | 34 | 38 | 41 | 44 | 53 | 64 | 68 | 72 | 79 |
|  | 45–54 | 36 | 38 | 41 | 45 | 55 | 65 | 72 | 77 | 84 |
|  | 55–64 | 33 | 37 | 40 | 44 | 53 | 65 | 73 | 78 | 89 |
|  | 65–74 | 33 | 37 | 40 | 45 | 54 | 65 | 71 | 76 | 84 |
|  | 75+ | 32 | 37 | 40 | 44 | 55 | 65 | 71 | 76 | 86 |

## 10.3 CKD Progression Validation

Once confident of the internal validity of demographics and risk factor assignment processes, we validated CKD progression. Validation of CKD progression is complicated by the fact that Kidney Disease Outcomes Quality Initiative (K/DOQI) stages are based on both albuminuria and glomerular filtration rate (GFR). We therefore initially considered albuminuria. We identified microalbuminuria incidence rates for persons with diabetes and macroalbuminuria transition rates for persons with hypertension and persons with diabetes. For all other micro and macro rates, we conducted our own analysis of NHANES data and developed a model process to duplicate microalbuminuria incidence and subsequent progression to macroalbuminuria (Table 10-4). Using this process, we were able to closely replicate NHANES prevalence rates for persons with neither hypertension nor diabetes. However, using the published rates for persons with hypertension or diabetes yields higher prevalence than found in NHANES data for these groups, and therefore the results for the total population are slightly above the NHANES values.

Table 10-4. Internal Validation of Albuminuria Prevalence

| Prevalence of Albuminuria | Persistent Microalbuminuria | |  | Macroalbuminuria | |
| --- | --- | --- | --- | --- | --- |
|  | NHANES | Model |  | NHANES | Model |
| Age |  |  |  |  |  |
| 39 | 3.3% | 4.0% |  | 0.5% | 0.7% |
| 55 | 5.9% | 6.8% |  | 1.5% | 1.8% |
| 73 | 14.5% | 16.9% |  | 3.6% | 3.8% |

Because of limited available data, it is not feasible to externally validate GFR reduction rates. However, accepting the accuracy of the model’s albuminuria incidence rates, the accuracy of GFR reduction rates should be reflected in CKD stage prevalence rates. The model’s default GFR process is based on the annual decrement values compiled in Boulware et al. (2003), reduced by a coefficient based on annual GFR decline observed in NHANES data. The value is randomized based on multipliers distributed in a triangular distribution from 0 to 2 upon incidence of macroalbuminuria. Adjusting model outputs to account for differences in population age structure yields CKD stage prevalence rates similar to those found in NHANES data by Coresh et al. (2007) (Table 10-5). The model overpredicts stages 1 and 2, although this has little effect on model outcomes. The model appears to achieve good agreement with stage 3. The model predicts relatively higher prevalence of stage 4 than found in NHANES data. However, NHANES may underrepresent persons with more advanced disease because it excludes individuals who are sick or institutionalized or may underrepresent older patients with lower GFR. Thus, we do not know to what extent, if any, the model is overpredicting prevalence of stage 4 CKD. Our overall estimate for stages 1 through 4 is approximately 15% higher than the NHANES values calculated by Coresh et al.

Table 10-5. External Validation of CKD Stage Prevalence Rates

| KDOQI Stage Prevalence (Person Years) | Stage 1 | Stage 2 | Stage 3 | Stage 4 | Stages 1–4 |
| --- | --- | --- | --- | --- | --- |
| Coresh et al. (2007) NHANES 1999–2004 | 0.018 | 0.032 | 0.077 | 0.004 | 0.130 |
| Age-adjusted model output | 0.030 | 0.038 | 0.075 | 0.007 | 0.150 |

Because of limited sample size and the exclusion of institutionalized patients, NHANES cannot be used to generate prevalence rates of stage 5 CKD. We can validate stage 5 prevalence rates by comparing the lifetime expected incidence of stage 5 to the lifetime incidence of ESRD. We estimated a 3.03% lifetime incidence of ESRD based on USRDS data (USRDS, 2006a) and census life tables (Arias, 2008). The current model predicts 4.71% lifetime incidence of stage 5 and 3.98% incidence of ESRD (Table 10-6). We found that many components of the model demonstrated good agreement with internal and external comparators (Tables 10‑4, 10‑5, and 10‑7); however, the lifetime incidence of stage 5 CKD in relative terms is higher than the estimated lifetime incidence of ESRD. Although a discrepancy remains between predicted stage 5 incidence and estimated ESRD incidence rates, we surmise that this could occur because it is likely that not all persons who reach stage 5 would be assigned ESRD status. This could arise if a person is not diagnosed, is too elderly or otherwise ill to be treated with dialysis or transplantation, or dies too quickly to be assigned to ESRD. One study of a dialysis population found that the median duration between referral and the beginning of dialysis was 11 months for individuals referred at stage 5 CKD (Navaneethan et al., 2007). Our assumption that agents must spend 1 year in stage 5 before initiating ESRD reduces the lifetime incidence of ESRD, but our estimates still appear to be higher than USRDS data. In the standard results, we made no effort to calibrate the model to force these values to match. However, overpredicting stage 5 incidence may yield a non-conservative model and thus could bias intervention outcomes. We conducted sensitivity analyses on several parameters that we considered may include uncertainty and/or may have a major impact on CKD progression. The goal of this analysis was not to conduct a full sensitivity analysis on input parameters but rather to explore options for model calibration to force a closer match between CKD stage 5 incidence and estimated ESRD incidence. Our calibration results were generally unsatisfactory in terms of external validation (we ended up with too few persons at earlier CKD stages); however, we found no significant impact on incremental cost-effectiveness ratios while including them.

Table 10-6. External Validation of Stage 5 Incidence

| Cumulative Incidence Stage5/ESRD |  |
| --- | --- |
| USRDS—ESRD | 0.0303 |
| Model—stage 5 | 0.0471 |
| Model-stage 5 after first year of ESRD mortality | 0.0398 |

Table 10-7. Selected Model Output

| Costs (mean, discounted 3%) | $149,672 |  |  |  |  |
| --- | --- | --- | --- | --- | --- |
| QALYs (mean, discounted 3%) | 17.829 |  |  |  |  |
| Life Years (mean) | 27.868 |  |  |  |  |
| **Proportion of Person Years in Each Stage** | **Total** | **30–39** | **40–59** | **60–69** | **70+** |
| Normal (share of person years) | 0.778 | 0.952 | 0.846 | 0.710 | 0.558 |
| Stage 1 (share of person years) | 0.034 | 0.019 | 0.041 | 0.040 | 0.032 |
| Stage 2 (share of person years) | 0.056 | 0.009 | 0.041 | 0.077 | 0.109 |
| Stage 3 (share of person years) | 0.115 | 0.020 | 0.068 | 0.153 | 0.254 |
| Stage 4 (share of person years) | 0.013 | 0.000 | 0.003 | 0.015 | 0.038 |
| Stage 5 (share of person years) | 0.004 | 0.000 | 0.001 | 0.005 | 0.010 |
| **Age-specific Prevalence** | **alive_40** | **alive_50** | **alive_60** | **alive_70** | **alive_80** |
| Normal (age prevalence) | 91.0% | 84.7% | 77.2% | 71.4% | 74.7% |
| Stage 1 (age prevalence) | 3.6% | 4.2% | 3.9% | 2.9% | 1.6% |
| Stage 2 (age prevalence) | 2.1% | 4.1% | 6.2% | 7.4% | 6.1% |
| Stage 3 (age prevalence) | 3.2% | 6.7% | 11.4% | 15.7% | 14.6% |
| Stage 4 (age prevalence) | 0.0% | 0.3% | 0.9% | 2.0% | 2.3% |
| Stage 5 (age prevalence) | 0.0% | 0.1% | 0.3% | 0.6% | 0.7% |
| **Stage Prevalence at End** |  |  | **Highest Stage Attained** | | |
| Normal | 0.1070 |  | Normal | 0.4727 |  |
| Stage 1 | 0.0060 |  | Stage1 | 0.0285 |  |
| Stage 2 | 0.0324 |  | Stage2 | 0.0999 |  |
| Stage 3 | 0.0792 |  | Stage3 | 0.2780 |  |
| Stage 4 | 0.0096 |  | Stage4 | 0.0738 |  |
| Stage 5 | 0.0013 |  | Stage5 | 0.0471 |  |
| Dead | 0.7645 |  |  |  |  |
| **CVD/CHD Events** |  |  | **Risk Factors** | | |
| CVD (lifetime incidence) | 0.605 |  | Diabetes (lifetime incidence) | | 0.324 |
| CHD (lifetime incidence) | 0.541 |  | HTN (lifetime incidence) | | 0.595 |
| CHD upon incident MI (lifetime incidence) | 0.204 |  | LVH (lifetime incidence) | | 0.400 |
| MI (lifetime incidence) | 0.299 |  | Smoker (lifetime incidence) | | 0.208 |
| Stroke (lifetime incidence) | 0.225 |  |  | |  |

References

Agency for Healthcare Research and Quality (AHRQ). 2004 state estimates on emergency department use. HCUP State Inpatient Database (SID) and State Emergency Department Databases (SEDD). http://hcupnet.ahrq.gov/. Accessed July 20, 2007.

Agency for Healthcare Research and Quality (AHRQ). 2005 national estimates on hospital use for all patients. HCUP Nationwide Inpatient Sample (NIS). http://hcupnet.ahrq.gov/. Accessed July 20, 2007.

Adler AI, Stevens RJ, Manley SE, et al. Development and progression of nephropathy in type 2 diabetes: the United Kingdom Prospective Diabetes Study (UKPDS 64). *Kidney International* 2003;63:225-232.

Agodoa LY, Appel L, Bakris GL et al. Effect of ramipril vs. Amlodipine on renal outcomes in hypertensive nephrosclerosis: a randomized controlled trial. *JAMA* 2001;85:2719-28.

Anderson KM, Odell PM, Wilson PWF, Kannnel WB. Cardiovascular disease risk profiles. *American Heart Journal* 1990;121:293-298.

Arias E. United States Life Tables, 2003. *National Vital Statistics Reports* 2006;54(14). http://www.cdc.gov/nchs/data/nvsr/nvsr54/nvsr54_14.pdf. Accessed July 30, 2007.

Boulware LE, Jaar BG, Tarver-Carr ME, Brancati FL, Powe NR. Screening for proteinuria in US adults: a cost-effectiveness analysis. *Journal of the American Medical Association* 2003;290(23):3101-3114.

Boulware LE, Troll MU, Jaar BG, Myers DI, Powe NR. Identification and referral of patients with progressive CKD: A national study. *Am J Kidney Dis.* 2006;48(2):192-204.

Bureau of Labor Statistics (BLS). Consumer Price Index. Available at: http://data.bls.gov/cgi-bin/surveymost?cu. Accessed July 20, 2007.

Centers for Medicare & Medicaid Services. 2007 Clinical Diagnostic Laboratory Fee Schedule—07CLAB. Available at: http://www.cms.hhs.gov/ClinicalLabFeeSched/
02_clinlab.asp#TopOfPage. Accessed July 20, 2007.

Centers for Medicare & Medicaid Services. Physician Fee Schedule Search, 2007. Available at: http://www.cms.hhs.gov/pfslookup/02_PFSsearch.asp. Accessed July 20, 2007.

Coresh J, Astor BC, Green T, Eknoyan G, Levey AS. Prevalence of chronic kidney disease and decreased kidney function in the adult US population: Third national health and nutrition examination survey. *American Journal of Kidney Diseases* 2003;41(1):1-12.

Coresh J, Byrd-Holt D, Astor BC, Briggs JP, Eggers PW, Lacher DA, Hostetter TH. Chronic kidney disease awareness, prevalence, and trends among US adults, 1999 to 2000. *Journal of the American Society of Nephrology* 2005;16:180-188.

Coresh J, Selvin E, Stevens L, Manzi J, Kusek J, Eggers P, Van Lente F, Levey A. Prevalence of chronic kidney disease in the United States. *Journal of the American Medical Association* 2007;298(17):2038-2047.

Cottone S, Nardi E, Mule G, Vadala A. Association between biomarkers of inflammation and left ventricular hypertrophy in moderate chronic kidney disease. *Clinical Nephrology* 2007 Apr;67(4):209-216.

Cowie CC, Rust KF, Bryd-Hold DD, Eberhardt MS, Flegal KM, Engelgau MM, Saydah SH, Williams DE, Geiss LS, Gregg EW. Prevalence of diabetes and impaired fasting glucose in adults in the U.S. Population: National Health and Nutrition Examination Survey 1999–2002. *Diabetes Care* 2006;29:1263–1268.

Drug Topics Redbook. Oradell, NJ: Medical Economics Co.; 2007.

Family Blood Pressure Program. Left Ventricular Hypertrophy. Available at: http://www.sph.uth.tmc.edu/hgc/fbpp/techSigLVH.htm. Accessed August 1, 2007.

Geiss LS, Pan L, Cadwell B, Gregg EW, Benjamin SM, Engelgau MM. Changes in incidence of diabetes in US adults, 1997-2003. *American Journal of Preventive Medicine* 2006;30(5):371-377.

Go AS, Chertow GM, Fan D, McCulloch CE, Hsu C. Chronic kidney disease and the risks of death, cardiovascular events, and hospitalization. *New England Journal of Medicine* 2004;351:1296-1305.

Gorodetskaya I, Zenios S, McCulloch CE, Bostrom A, Hsu C, Bindman AB, Go AS, Chertow GM. Health-related quality of life and estimates of utility in chronic kidney disease. *Kidney International* 2005;68:2801-2808.

Hunink MG, Goldman L, Tosteson AN, MIttleman MA, Goldman PA, Williams LW, Tsevat J, Weinstein MC. The recent decline in mortality from coronary heart disease, 1980-1990. the effect of secular trends in risk factors and treatment. *Journal of the American Medical Association* 1997;277(7):535-542.

Hunsicker LG, Adler S, Caggiula A, Englad BK, Greene T, Kusek JW, Roger NL, Teschan PE. Predictors of the progression of renal disease in the Modification of Diet in Renal Disease Study. *Kidney International* 1997;51:1908-1919.

Jafar TH, Schmid CH, Landa M, Giatras I, Toto R, et al. Angiotensin-converting enzyme inhibitors and progresson of nondiabetic renal disease: A meta-analysis of patient-level data. *Annals of Internal Medicine* 2001;135:73-87.

Kiberd BA, Clase CM. Cumulative risk for developing end-stage renal disease in the US population. *Journal of the American Society of Nephrology* 2002;13:1635-1644.

Levey AS, Coresh J, Balk E, Kausz AT, Levin A, et al. National Kidney Foundation practice guidelines for chronic kidney disease: evaluation, classification, and stratification. *Annals of Internal Medicine* 2003;139:137-147.

Levey AS, Coresh J, Greene T, et al. Using standardized serum creatinine values in the modification of diet in renal disease study equation for estimating glomerular filtration rate. *Annals of Internal Medicine* 2006;145:247-254.

Levey AS, Greene T, Kusek JW, Beck GL, MDRD Study Group. A simplified equation to predict glomerular filtration rate from serum creatinine. *Journal of the American Society of Nephrology* 2000;11:155A.

Lewis EJ, Hunsicker LG, Clarke WR, Berl T, Pohl MA, Lewis JB, Ritz E, Atkins RC, Rohde R, Raz I. Renoprotective effect of angiotensin-receptor antagonist irbesartan in patients with nephropathy due to type 2 diabetes. *New England Journal of Medicine* 2001;345:851-860.

Mann JF, Gerstein HC, Yi QL, et al. Development of renal disease in people at high cardiovascular risk: result of the HOPE randomized study. *Journal of the American Society of Nephrology* 2003;14:641-647.

Maurice E, Trosclair A, Merritt R. Cigarette smoking among adults—United States, 2004. *MMWR* 2005;54(44):1121-1124.

Meenan RT, Saha S, Chou R, et al. Cost-effectiveness of echocardiography to identify intracardiac thrombus among patients with first stroke or transient ischemic attack. *Medical Decision Making* 2007;27:161-177.

Nakao N, Yoshimura A, Morita H, Takada M, Kayano T, Ideura T. Combination treatment of angiotensin-II receptor blocker and angiotensin-converting enzyme inhibitor in non-diabetic renal disease (COOPERATE): a randomised trial. *Lancet* 2003;361(9352): 117-124.

National Center for Health Statistics (NCHS). Worktable 210R: Death rates for 113 selected causes, alcohol-induced causes, drug-induced causes, and injury by firearms by 5-year age groups, race, and sex: United States 2003. March 2006. Available at: http://www.cdc.gov/nchs/data/statab/Mortfinal2003_worktable210r.pdf. Accessed July 30, 2007.

National Institutes of Health. *Third Report of the National Cholesterol Education Program Expert Panel on Detection, Evaluation, and Treatment of High Blood Cholesterol in Adults (Adult Treatment Panel III)*. NIH Publication 01-3670. Bethesda, MD: National Institutes of Health; 2001.

National Kidney Foundation. K/DOQI clinical practice guidelines and clinical practice recommendations for diabetes and chronic kidney disease. *American Journal of Kidney Diseases* 2007;49:S12-S154.

National Kidney Foundation. Guideline 5: assessment of proteinuria. K/DOQI Clinical practice guidelines for chronic kidney disease: evaluation, classification and stratification. *American Journal of Kidney Diseases* 2002;39(suppl 1):S1-S000. Available at: http://www.kidney.org/professionals/kdoqi/guidelines_ckd/
p5_lab_g5.htm.

Navaneethan SD, Nigwekar S, Sengodan M, et al. Referral to nephrologists for chronic kidney disease care: Is non-diabetic kidney disease ignored. *Nephron Clin Pract.* 2007;106:c113-c118.

Nease RF, Kneeland T, O’Connor GT, et al. Variation in patient utilities for outcomes of the management of chronic stable angina. *Journal of the American Medical Association* 1995;273(15):1185-1190.

O’Hare A, Bertenthal D, Covinski K, Landenfald CS, Sen S. Mortality risk stratification in chronic kidney disease: one size for all ages? *Journal of the American Society of Nephrology* 2006;17:846-853.

Paoletti E, Bellino D, Cassottana P, Rolla D, Cannella G. Left ventricular hypertrophy in nondiabetic predialysis CKD. *American Journal of Kidney Diseases* 2005;46(2):320-327.

Pogio ED, Wang X, Green T, et al. Performance of the modification of diet in renal disease and Cockcroft-Gault equations in the estimation of GFR in health and in chronic kidney disease. *Journal of the American Society of Nephrology* 2005;16:459-466.

Rodby RA, Chiou CF, Borenstein J, et al. The cost-effectiveness of irbesartan in the treatment of hypertensive patients with type 2 diabetic nephropathy. *Clinical Therapeutics* 2003;25:2103–2119.

Ruggenenti P, Perna A, Gherardi G, et al. Renoprotective properties of ACE-inhibition in non-diabetic nephropathies with non-nephrotic proteinuria. *Lancet* 1999;354:359-364.

Ruggenenti P, Perna A, Mosconi L, et al. (GISEN group) Randomised placebo-controlled trial of effect of ramipril on decline in glomerular filtration rate and risk of terminal renal failure in proteinuric, non-diabetic nephropathy. *Lancet* 1997;349:1857-1863.

Rule AD, Lanson TS, Bergstralh EJ, et al. Using serum creatinine to estimate glomerular filtration rate: accuracy in good health and in chronic kidney disease. *Annals of Internal Medicine* 2004;141:929-937.

Sacco RL, Shi T, Zamanillo MC, Kargman DE. Predictors of mortality and recurrence after hospitalized cerebral infarction in an urban community: the Northern Manhattan Stroke Study. *Neurology* 1994;44:626-634.

Sarafidis PA, Riehle J, Bogojevic Z, et al. A comparative evaluation of various methods for microalbuminuria screening. *Am J Nephrol.* 2008;28(2):324-329.

Saydah S, Eberhardt M, Rios-Burrows N, Williams D, Geiss L, Dorsey R. Prevalence of chronic kidney disease and associated risk factors—United States 1999-2004. *Morbidity and Mortality Weekly Report* 2007;56(8):161-165.

Smith DH, Nichols GA, Gullion CM, Johnson ES, Keith D. Predicting costs of care in chronic kidney disease: the role of comorbid conditions. *The Internet Journal of Nephrology* 2007;4(1). Available at: http://www.ispub.com/ostia/index.php?xmlFilePath=journals/
ijne/vol4n1/cost.xml. Accessed October 31, 2007.

Strippoli GF, Craig M, Deeks JJ, Schena FP, Craig JC. Effects of Angiotensin converting enzyme inhibitors and Angiotensin II receptor antagonists on mortality and renal outcomes in diabetic nephropathy: Systematic review. *BMJ* 2004;329:828.

Tsevat J, Goldman L, Soukup JR, et al. Stability of time-tradeoff utilities in survivors of myocardial infarction. *Medical Decision Making* 1993;13(2):161-165.

United States Renal Data System (USRDSa). *USRDS 2006 Annual Data Report:* Incidence of Reported ESRD Reference Tables. Bethesda, MD: National Institutes of Health, National Institute of Diabetes and Digestive and Kidney Diseases; 2006. Table A.2, page 10, http://www.usrds.org/2006/ref/A_incidence_06.pdf. Accessed July 30, 2009.

United States Renal Data System (USRDSb). USRDS 2006 Annual Data Report: Atlas of Chronic Kidney Disease and End-Stage Renal Disease in the United States, National Institutes of Health, National Institute of Diabetes and Digestive and Kidney Diseases, Bethesda, MD; 2006. Figure 11.3, page 208, http://www.usrds.org/2006/pdf/01_ckd_06.pdf. Accessed July 30, 2009.

United States Renal Data System (USRDSc). *USRDS 2006 Annual Data Report:* Incidence of Reported ESRD Reference Tables. Bethesda, MD: National Institutes of Health, National Institute of Diabetes and Digestive and Kidney Diseases; 2006. Table A.1, page 6, http://www.usrds.org/2006/ref/A_incidence_06.pdf. Accessed July 30, 2009.

United States Renal Data System (USRDSd). *USRDS 2006 Annual Data Report:* Prevalence of Reported ESRD Reference Tables. Bethesda, MD: National Institutes of Health, National Institute of Diabetes and Digestive and Kidney Diseases; 2006. Table B.1, page 40, http://www.usrds.org/2006/ref/B_prevalence_06.pdf. Accessed July 30, 2009.

U.S. Renal Data System (USRDSe). USRDS 2006 Annual Data Report: Atlas of Chronic Kidney Disease and End-Stage Renal Disease in the United States, National Institutes of Health, National Institute of Diabetes and Digestive and Kidney Diseases, Bethesda, MD; 2006. Figure 1.8, page 53, http://www.usrds.org/2006/pdf/01_ckd_06.pdf. Accessed July 30, 2009.

United States Renal Data System (USRDSa). *USRDS 2008 Annual Data Report:* Volume 3 Reference Tables on End-Stage Renal Disease. Bethesda, MD: National Institutes of Health, National Institute of Diabetes and Digestive and Kidney Diseases; 2008. Table B.1, page 31, http://www.usrds.org/2008/ref/B_Prevalence_08.pdf. Accessed July 30, 2009.

United States Renal Data System (USRDSb). *USRDS 2008 Annual Data Report:* Volume 2 Atlas of End-Stage Renal Disease. Bethesda, MD: National Institutes of Health, National Institute of Diabetes and Digestive and Kidney Diseases; 2008. Figure 11.1, page 177, <http://www.usrds.org/2008/pdf/V2_11_2008.pdf>. Accessed July 30, 2009.

United States Renal Data System (USRDSc). *USRDS 2008 Annual Data Report:* Volume 1 Atlas of Chronic Kidney Disease. Bethesda, MD: National Institutes of Health, National Institute of Diabetes and Digestive and Kidney Diseases; 2008. Figure 5.2, page 84, http://www.usrds.org/2008/pdf/V1_05_2008.pdf. Accessed July 30, 2009.

United States Renal Data System (USRDSd). RenDER Renal Data Extraction and Referencing System. ESRD death rate per 1,000 patient years. Available at: http://www.usrds.org/odr/xrender_home.asp. Accessed June 18, 2007.

U.S. Census Bureau. Table 4: Annual Estimates of the Population by Sex and Age for the United States: April 1, 2000 to July 1, 2007 (NC-EST2007-04-WANH, NC-EST2007-04-BAC, NC-EST2007-04-Hisp). Population Division, U.S. Census Bureau. Release Date: May 1, 2008.

Weiner DE, Tabatabai S, Tighiouart H, Elsayed E, Bansal N, Griffith J, Salem DN, Levey AS, Sarnak MJ. Cardiovascular outcomes and all-cause mortality: exploring the interaction between CKD and cardiovascular disease. *American Journal of Kidney Disease* 2006;48:392-401.

Weinstein MC, O’Brien B, Hornberger J, et al. Principles of good practice for decision analytic modeling in health-care evaluation. Report of the ISPOR task force on good research practices—modeling studies. *Value in Health* 2003;6:9-17.

1. Data Inputs

Table A-1. Age 30 Demographics

| **Race/Sex Group** | **Age 30 Prevalence** |
| --- | --- |
| White men | 0.36522 |
| African American men | 0.06045 |
| Hispanic men | 0.07758 |
| White women | 0.35729 |
| African American women | 0.06723 |
| Hispanic women | 0.07224 |

Table A-2a. Microalbuminuria Incidence, Non-African American (Age 30 Prevalence)

|  | **Neither** | | **Hypertension** | | **Diabetes** | |
| --- | --- | --- | --- | --- | --- | --- |
| **Age** | **Men** | **Women** | **Men** | **Women** | **Men** | **Women** |
| 30 | 0.002085 | 0.006918 | 0.010476 | 0.033855 | 0.009675 | 0.071624 |
| 31 | 0.00545 | 0.005459 | 0.006129 | 0.006204 | 0.009705 | 0.008158 |
| 32 | 0.005534 | 0.005534 | 0.006242 | 0.006269 | 0.009735 | 0.008271 |
| 33 | 0.005619 | 0.005609 | 0.006355 | 0.006335 | 0.009765 | 0.008385 |
| 34 | 0.005704 | 0.005684 | 0.006468 | 0.006401 | 0.009796 | 0.008501 |
| 35 | 0.005789 | 0.00576 | 0.006583 | 0.006467 | 0.009827 | 0.008619 |
| 36 | 0.005875 | 0.005835 | 0.006698 | 0.006534 | 0.009858 | 0.008738 |
| 37 | 0.005961 | 0.005912 | 0.006814 | 0.006602 | 0.00989 | 0.008858 |
| 38 | 0.006048 | 0.005988 | 0.006931 | 0.006669 | 0.009922 | 0.00898 |
| 39 | 0.006135 | 0.006065 | 0.007049 | 0.006738 | 0.009955 | 0.009103 |
| 40 | 0.002239 | 0.002159 | 0.003184 | 0.002823 | 0.006004 | 0.005245 |
| 41 | 0.002327 | 0.002237 | 0.003304 | 0.002892 | 0.006037 | 0.005372 |
| 42 | 0.002416 | 0.002315 | 0.003424 | 0.002962 | 0.006071 | 0.005501 |
| 43 | 0.002505 | 0.002394 | 0.003546 | 0.003032 | 0.006105 | 0.005631 |
| 44 | 0.002595 | 0.002473 | 0.00367 | 0.003103 | 0.006139 | 0.005764 |
| 45 | 0.002685 | 0.002553 | 0.003794 | 0.003174 | 0.006174 | 0.005899 |
| 46 | 0.002776 | 0.002633 | 0.003919 | 0.003247 | 0.006209 | 0.006036 |
| 47 | 0.002868 | 0.002714 | 0.004046 | 0.003319 | 0.006245 | 0.006174 |
| 48 | 0.00296 | 0.002795 | 0.004175 | 0.003393 | 0.006281 | 0.006316 |
| 49 | 0.003053 | 0.002877 | 0.004304 | 0.003466 | 0.006318 | 0.006459 |
| 50 | 0.003147 | 0.00296 | 0.004436 | 0.003541 | 0.006355 | 0.006605 |
| 51 | 0.003242 | 0.003043 | 0.004568 | 0.003616 | 0.006392 | 0.006754 |
| 52 | 0.003337 | 0.003127 | 0.004703 | 0.003693 | 0.00643 | 0.006905 |
| 53 | 0.003433 | 0.003212 | 0.004839 | 0.003769 | 0.006468 | 0.007059 |

(continued)

Table A-2a. Microalbuminuria Incidence, Non-African American (Age 30 Prevalence) (continued)

|  | **Neither** | | **Hypertension** | | **Diabetes** | |
| --- | --- | --- | --- | --- | --- | --- |
| **Age** | **Men** | **Women** | **Men** | **Women** | **Men** | **Women** |
| 54 | 0.003531 | 0.003297 | 0.004977 | 0.003847 | 0.006507 | 0.007216 |
| 55 | 0.003629 | 0.003383 | 0.005117 | 0.003925 | 0.006547 | 0.007375 |
| 56 | 0.004326 | 0.004069 | 0.005857 | 0.004603 | 0.007185 | 0.008137 |
| 57 | 0.004426 | 0.004156 | 0.006001 | 0.004683 | 0.007225 | 0.008303 |
| 58 | 0.004527 | 0.004245 | 0.006147 | 0.004764 | 0.007266 | 0.008472 |
| 59 | 0.00463 | 0.004334 | 0.006295 | 0.004846 | 0.007307 | 0.008645 |
| 60 | 0.004733 | 0.004425 | 0.006445 | 0.004928 | 0.007349 | 0.008821 |
| 61 | 0.004837 | 0.004516 | 0.006597 | 0.005012 | 0.007392 | 0.009001 |
| 62 | 0.004943 | 0.004608 | 0.006752 | 0.005097 | 0.007435 | 0.009185 |
| 63 | 0.00505 | 0.004701 | 0.00691 | 0.005182 | 0.007479 | 0.009373 |
| 64 | 0.005158 | 0.004795 | 0.00707 | 0.005269 | 0.007523 | 0.009565 |
| 65 | 0.005267 | 0.00489 | 0.007233 | 0.005357 | 0.007568 | 0.009762 |
| 66 | 0.005378 | 0.004987 | 0.007399 | 0.005445 | 0.007613 | 0.009963 |
| 67 | 0.00549 | 0.005084 | 0.007568 | 0.005535 | 0.007659 | 0.010169 |
| 68 | 0.005603 | 0.005183 | 0.00774 | 0.005626 | 0.007706 | 0.01038 |
| 69 | 0.005719 | 0.005283 | 0.007915 | 0.005719 | 0.007753 | 0.010596 |
| 70 | 0.005835 | 0.005384 | 0.008093 | 0.005812 | 0.007801 | 0.010818 |
| 71 | 0.005954 | 0.005486 | 0.008275 | 0.005907 | 0.007849 | 0.011046 |
| 72 | 0.006074 | 0.005589 | 0.008461 | 0.006003 | 0.007899 | 0.011279 |
| 73 | 0.006195 | 0.005694 | 0.008651 | 0.006101 | 0.007949 | 0.011519 |
| 74 | 0.006319 | 0.005801 | 0.008844 | 0.006199 | 0.007999 | 0.011765 |
| 75 | 0.006444 | 0.005909 | 0.009042 | 0.0063 | 0.008051 | 0.012018 |
| 76 | 0.006572 | 0.006018 | 0.009244 | 0.006402 | 0.008103 | 0.012278 |
| 77 | 0.006701 | 0.006129 | 0.00945 | 0.006505 | 0.008156 | 0.012545 |
| 78 | 0.006832 | 0.006242 | 0.009662 | 0.00661 | 0.00821 | 0.012821 |
| 79 | 0.006966 | 0.006356 | 0.009878 | 0.006716 | 0.008264 | 0.013105 |
| 80 | 0.007102 | 0.006472 | 0.010099 | 0.006825 | 0.00832 | 0.013398 |
| 81 | 0.00724 | 0.006589 | 0.010326 | 0.006934 | 0.008376 | 0.013699 |
| 82 | 0.00738 | 0.006709 | 0.010559 | 0.007046 | 0.008433 | 0.014011 |
| 83 | 0.007523 | 0.00683 | 0.010797 | 0.00716 | 0.008491 | 0.014332 |
| 84 | 0.007669 | 0.006954 | 0.011041 | 0.007275 | 0.008549 | 0.014665 |
| 85 | 0.007817 | 0.007079 | 0.011292 | 0.007393 | 0.008609 | 0.015008 |
| 86 | 0.007968 | 0.007207 | 0.01155 | 0.007512 | 0.00867 | 0.015364 |
| 87 | 0.008122 | 0.007337 | 0.011815 | 0.007634 | 0.008731 | 0.015733 |
| 88 | 0.008279 | 0.007469 | 0.012088 | 0.007758 | 0.008794 | 0.016114 |
| 89 | 0.008439 | 0.007603 | 0.012368 | 0.007884 | 0.008858 | 0.01651 |
| 90 | 0.008602 | 0.00774 | 0.012657 | 0.008012 | 0.008922 | 0.016921 |

Table A-2b. Microalbuminuria Incidence, African American (Age 30 Prevalence)

|  | **Neither** | | **Hypertension** | | **Diabetes** | |
| --- | --- | --- | --- | --- | --- | --- |
| **Age** | **Men** | **Women** | **Men** | **Women** | **Men** | **Women** |
| 30 | 0.033996 | 0.044391 | 0.068151 | 0.022455 | 0.181056 | 0.178404 |
| 31 | 0.005015 | 0.004942 | 0.00693 | 0.005704 | 0.00995 | 0.008759 |
| 32 | 0.005058 | 0.004956 | 0.007025 | 0.00582 | 0.009956 | 0.008681 |
| 33 | 0.005101 | 0.00497 | 0.007122 | 0.005937 | 0.009961 | 0.008601 |
| 34 | 0.005144 | 0.004983 | 0.00722 | 0.006055 | 0.009965 | 0.00852 |
| 35 | 0.005187 | 0.004997 | 0.007318 | 0.006174 | 0.00997 | 0.008438 |
| 36 | 0.005231 | 0.005011 | 0.007418 | 0.006294 | 0.009974 | 0.008354 |
| 37 | 0.005275 | 0.005025 | 0.007519 | 0.006415 | 0.009979 | 0.008269 |
| 38 | 0.005319 | 0.005039 | 0.007621 | 0.006537 | 0.009983 | 0.008183 |
| 39 | 0.005363 | 0.005053 | 0.007725 | 0.00666 | 0.009986 | 0.008095 |
| 40 | 0.005243 | 0.004903 | 0.007664 | 0.006619 | 0.009825 | 0.00784 |
| 41 | 0.005288 | 0.004917 | 0.00777 | 0.006745 | 0.009828 | 0.00775 |
| 42 | 0.005333 | 0.004931 | 0.007878 | 0.006872 | 0.009831 | 0.007658 |
| 43 | 0.005378 | 0.004946 | 0.007986 | 0.007 | 0.009834 | 0.007564 |
| 44 | 0.005424 | 0.00496 | 0.008097 | 0.007129 | 0.009837 | 0.00747 |
| 45 | 0.00547 | 0.004975 | 0.008208 | 0.00726 | 0.009839 | 0.007374 |
| 46 | 0.005516 | 0.004989 | 0.008322 | 0.007393 | 0.009841 | 0.007276 |
| 47 | 0.005563 | 0.005004 | 0.008436 | 0.007527 | 0.009843 | 0.007178 |
| 48 | 0.00561 | 0.005019 | 0.008553 | 0.007663 | 0.009845 | 0.007078 |
| 49 | 0.005657 | 0.005034 | 0.008671 | 0.007801 | 0.009846 | 0.006976 |
| 50 | 0.005705 | 0.005048 | 0.008791 | 0.00794 | 0.009847 | 0.006874 |
| 51 | 0.005753 | 0.005063 | 0.008913 | 0.008081 | 0.009848 | 0.00677 |
| 52 | 0.005801 | 0.005078 | 0.009037 | 0.008224 | 0.009848 | 0.006666 |
| 53 | 0.00585 | 0.005094 | 0.009163 | 0.00837 | 0.009848 | 0.00656 |
| 54 | 0.005899 | 0.005109 | 0.009291 | 0.008517 | 0.009848 | 0.006453 |
| 55 | 0.005948 | 0.005124 | 0.009421 | 0.008667 | 0.009848 | 0.006344 |
| 56 | 0.004011 | 0.003153 | 0.007566 | 0.006832 | 0.00786 | 0.004248 |
| 57 | 0.004061 | 0.003168 | 0.007701 | 0.006986 | 0.007859 | 0.004138 |
| 58 | 0.004112 | 0.003184 | 0.007837 | 0.007143 | 0.007858 | 0.004027 |
| 59 | 0.004163 | 0.003199 | 0.007977 | 0.007302 | 0.007856 | 0.003914 |
| 60 | 0.004214 | 0.003215 | 0.008118 | 0.007464 | 0.007854 | 0.003801 |
| 61 | 0.004266 | 0.003231 | 0.008263 | 0.007629 | 0.007852 | 0.003687 |
| 62 | 0.004318 | 0.003247 | 0.00841 | 0.007797 | 0.007849 | 0.003572 |
| 63 | 0.004371 | 0.003263 | 0.00856 | 0.007967 | 0.007846 | 0.003456 |
| 64 | 0.004424 | 0.003279 | 0.008713 | 0.008142 | 0.007842 | 0.003339 |
| 65 | 0.004478 | 0.003295 | 0.008869 | 0.008319 | 0.007839 | 0.003222 |
| 66 | 0.004532 | 0.003311 | 0.009028 | 0.0085 | 0.007834 | 0.003103 |

(continued)

Table A-2b. Microalbuminuria Incidence, African American (Age 30 Prevalence) (continued)

|  | **Neither** | | **Hypertension** | | **Diabetes** | |
| --- | --- | --- | --- | --- | --- | --- |
| **Age** | **Men** | **Women** | **Men** | **Women** | **Men** | **Women** |
| 67 | 0.004587 | 0.003328 | 0.009191 | 0.008684 | 0.00783 | 0.002984 |
| 68 | 0.004642 | 0.003344 | 0.009357 | 0.008872 | 0.007825 | 0.002865 |
| 69 | 0.004698 | 0.003361 | 0.009526 | 0.009064 | 0.00782 | 0.002745 |
| 70 | 0.004754 | 0.003377 | 0.009699 | 0.00926 | 0.007814 | 0.002624 |
| 71 | 0.004811 | 0.003394 | 0.009876 | 0.009461 | 0.007808 | 0.002502 |
| 72 | 0.004869 | 0.003411 | 0.010057 | 0.009665 | 0.007801 | 0.00238 |
| 73 | 0.004927 | 0.003428 | 0.010242 | 0.009875 | 0.007794 | 0.002258 |
| 74 | 0.004986 | 0.003445 | 0.010432 | 0.010089 | 0.007787 | 0.002135 |
| 75 | 0.005045 | 0.003462 | 0.010626 | 0.010308 | 0.007779 | 0.002012 |
| 76 | 0.005105 | 0.003479 | 0.010824 | 0.010533 | 0.007771 | 0.001889 |
| 77 | 0.005165 | 0.003497 | 0.011028 | 0.010763 | 0.007762 | 0.001765 |
| 78 | 0.005227 | 0.003514 | 0.011236 | 0.010999 | 0.007753 | 0.001641 |
| 79 | 0.005288 | 0.003532 | 0.01145 | 0.011241 | 0.007743 | 0.001517 |
| 80 | 0.005351 | 0.003549 | 0.01167 | 0.011489 | 0.007733 | 0.001393 |
| 81 | 0.005414 | 0.003567 | 0.011895 | 0.011744 | 0.007722 | 0.001269 |
| 82 | 0.005478 | 0.003585 | 0.012127 | 0.012006 | 0.007711 | 0.001144 |
| 83 | 0.005543 | 0.003603 | 0.012365 | 0.012275 | 0.007699 | 0.00102 |
| 84 | 0.005609 | 0.003621 | 0.012609 | 0.012552 | 0.007687 | 0.000896 |
| 85 | 0.005675 | 0.00364 | 0.012861 | 0.012838 | 0.007674 | 0.000772 |
| 86 | 0.005742 | 0.003658 | 0.013119 | 0.013131 | 0.007661 | 0.000647 |
| 87 | 0.00581 | 0.003676 | 0.013386 | 0.013434 | 0.007647 | 0.000524 |
| 88 | 0.005879 | 0.003695 | 0.01366 | 0.013745 | 0.007633 | 0.0004 |
| 89 | 0.005949 | 0.003714 | 0.013943 | 0.014067 | 0.007618 | 0.000277 |
| 90 | 0.006019 | 0.003733 | 0.014235 | 0.014399 | 0.007603 | 0.000153 |

Table A-3a. Micro- to Macroalbuminuria Transition Probabilities, Non-African American (Age 30 Macro Prevalence)

|  | **Neither** | | **Hypertension** | | **Diabetes** | |
| --- | --- | --- | --- | --- | --- | --- |
| **Age** | **Men** | **Women** | **Men** | **Women** | **Men** | **Women** |
| 30 | 0.000011 | 0.000011 | 0.000011 | 0.000011 | 0.000011 | 0.000011 |
| 31 | 0.046278 | 0.022149 | 0.056452 | 0.034981 | 0.078391 | 0.037779 |
| 32 | 0.046278 | 0.022149 | 0.056452 | 0.034981 | 0.078391 | 0.037779 |
| 33 | 0.046278 | 0.022149 | 0.056452 | 0.034981 | 0.078391 | 0.037779 |
| 34 | 0.046278 | 0.022149 | 0.056452 | 0.034981 | 0.078391 | 0.037779 |
| 35 | 0.046278 | 0.022149 | 0.056452 | 0.034981 | 0.078391 | 0.037779 |
| 36 | 0.046278 | 0.022149 | 0.056452 | 0.034981 | 0.078391 | 0.037779 |
| 37 | 0.046278 | 0.022149 | 0.056452 | 0.034981 | 0.078391 | 0.037779 |
| 38 | 0.046278 | 0.022149 | 0.056452 | 0.034981 | 0.078391 | 0.037779 |
| 39 | 0.046278 | 0.022149 | 0.056452 | 0.034981 | 0.078391 | 0.037779 |
| 40 | 0.00595 | 0.0024 | 0.028699 | 0.007252 | 0.017079 | 0.037976 |
| 41 | 0.00595 | 0.0024 | 0.028699 | 0.007252 | 0.017079 | 0.037976 |
| 42 | 0.00595 | 0.0024 | 0.028699 | 0.007252 | 0.017079 | 0.037976 |
| 43 | 0.00595 | 0.0024 | 0.028699 | 0.007252 | 0.019538 | 0.037976 |
| 44 | 0.00595 | 0.0024 | 0.028699 | 0.007252 | 0.019538 | 0.037976 |
| 45 | 0.00595 | 0.0024 | 0.028699 | 0.007252 | 0.019538 | 0.037976 |
| 46 | 0.00595 | 0.0024 | 0.028699 | 0.007252 | 0.019538 | 0.037976 |
| 47 | 0.00595 | 0.0024 | 0.028699 | 0.007252 | 0.019538 | 0.037976 |
| 48 | 0.00595 | 0.0024 | 0.028699 | 0.007252 | 0.019538 | 0.037976 |
| 49 | 0.00595 | 0.0024 | 0.028699 | 0.007252 | 0.019538 | 0.037976 |
| 50 | 0.00595 | 0.0024 | 0.028699 | 0.007252 | 0.019538 | 0.037976 |
| 51 | 0.00595 | 0.0024 | 0.028699 | 0.007252 | 0.019538 | 0.037976 |
| 52 | 0.00595 | 0.0024 | 0.028699 | 0.007252 | 0.019538 | 0.037976 |
| 53 | 0.00595 | 0.0024 | 0.028699 | 0.007252 | 0.019538 | 0.037976 |
| 54 | 0.00595 | 0.0024 | 0.028699 | 0.007252 | 0.019538 | 0.037976 |
| 55 | 0.00595 | 0.0024 | 0.028699 | 0.007252 | 0.019538 | 0.037976 |
| 56 | 0.012844 | 0.001737 | 0.006563 | 0.009467 | 0.019686 | 0.010385 |
| 57 | 0.012844 | 0.001737 | 0.006563 | 0.009467 | 0.019686 | 0.010385 |
| 58 | 0.012844 | 0.001737 | 0.006563 | 0.009467 | 0.014297 | 0.010385 |
| 59 | 0.012844 | 0.001737 | 0.006563 | 0.009467 | 0.014297 | 0.010385 |
| 60 | 0.012844 | 0.001737 | 0.006563 | 0.009467 | 0.014297 | 0.010385 |
| 61 | 0.012844 | 0.001737 | 0.006563 | 0.009467 | 0.014297 | 0.010385 |
| 62 | 0.012844 | 0.001737 | 0.006563 | 0.009467 | 0.014297 | 0.010385 |
| 63 | 0.012844 | 0.001737 | 0.006563 | 0.009467 | 0.014297 | 0.010385 |
| 64 | 0.012844 | 0.001737 | 0.006563 | 0.009467 | 0.014297 | 0.010385 |
| 65 | 0.012844 | 0.001737 | 0.006563 | 0.009467 | 0.014297 | 0.010385 |
| 66 | 0.012844 | 0.001737 | 0.006563 | 0.009467 | 0.014297 | 0.010385 |
| 67 | 0.012844 | 0.001737 | 0.006563 | 0.009467 | 0.014297 | 0.010385 |

(continued)

Table A-3a. Micro- to Macroalbuminuria Transition Probabilities, Non-African American (Age 30 Macro Prevalence) (continued)

|  | **Neither** | | **Hypertension** | | **Diabetes** | |
| --- | --- | --- | --- | --- | --- | --- |
| **Age** | **Men** | **Women** | **Men** | **Women** | **Men** | **Women** |
| 68 | 0.012844 | 0.001737 | 0.006563 | 0.009467 | 0.014297 | 0.010385 |
| 69 | 0.012844 | 0.001737 | 0.006563 | 0.009467 | 0.014297 | 0.010385 |
| 70 | 0.012844 | 0.001737 | 0.006563 | 0.009467 | 0.014297 | 0.010385 |
| 71 | 0.012844 | 0.001737 | 0.006563 | 0.009467 | 0.014297 | 0.010385 |
| 72 | 0.012844 | 0.001737 | 0.006563 | 0.009467 | 0.014297 | 0.010385 |
| 73 | 0.012844 | 0.001737 | 0.006563 | 0.009467 | 0.014297 | 0.010385 |
| 74 | 0.012844 | 0.001737 | 0.006563 | 0.009467 | 0.014297 | 0.010385 |
| 75 | 0.012844 | 0.001737 | 0.006563 | 0.009467 | 0.014297 | 0.010385 |
| 76 | 0.012844 | 0.001737 | 0.006563 | 0.009467 | 0.014297 | 0.010385 |
| 77 | 0.012844 | 0.001737 | 0.006563 | 0.009467 | 0.014297 | 0.010385 |
| 78 | 0.012844 | 0.001737 | 0.006563 | 0.009467 | 0.014297 | 0.010385 |
| 79 | 0.012844 | 0.001737 | 0.006563 | 0.009467 | 0.014297 | 0.010385 |
| 80 | 0.012844 | 0.001737 | 0.006563 | 0.009467 | 0.014297 | 0.010385 |
| 81 | 0.012844 | 0.001737 | 0.006563 | 0.009467 | 0.014297 | 0.010385 |
| 82 | 0.012844 | 0.001737 | 0.006563 | 0.009467 | 0.014297 | 0.010385 |
| 83 | 0.012844 | 0.001737 | 0.006563 | 0.009467 | 0.014297 | 0.010385 |
| 84 | 0.012844 | 0.001737 | 0.006563 | 0.009467 | 0.014297 | 0.010385 |
| 85 | 0.012844 | 0.001737 | 0.006563 | 0.009467 | 0.014297 | 0.010385 |
| 86 | 0.012844 | 0.001737 | 0.006563 | 0.009467 | 0.014297 | 0.010385 |
| 87 | 0.012844 | 0.001737 | 0.006563 | 0.009467 | 0.014297 | 0.010385 |
| 88 | 0.012844 | 0.001737 | 0.006563 | 0.009467 | 0.014297 | 0.010385 |
| 89 | 0.012844 | 0.001737 | 0.006563 | 0.009467 | 0.014297 | 0.010385 |
| 90 | 0.012844 | 0.001737 | 0.006563 | 0.009467 | 0.014297 | 0.010385 |

Table A-3b. Micro- to Macroalbuminuria Transition Probabilities, African American (Age 30 Macro Prevalence)

|  | **Neither** | | **Hypertension** | | **Diabetes** | |
| --- | --- | --- | --- | --- | --- | --- |
| **Age** | **Men** | **Women** | **Men** | **Women** | **Men** | **Women** |
| 30 | 0.032463 | 0.032463 | 0.032463 | 0.032463 | 0.032463 | 0.032463 |
| 31 | 0.07873 | 0.0546 | 0.088904 | 0.067432 | 0.110843 | 0.070231 |
| 32 | 0.07873 | 0.0546 | 0.088904 | 0.067432 | 0.110843 | 0.070231 |
| 33 | 0.07873 | 0.0546 | 0.088904 | 0.067432 | 0.110843 | 0.070231 |
| 34 | 0.07873 | 0.0546 | 0.088904 | 0.067432 | 0.110843 | 0.070231 |
| 35 | 0.07873 | 0.0546 | 0.088904 | 0.067432 | 0.110843 | 0.070231 |
| 36 | 0.07873 | 0.0546 | 0.088904 | 0.067432 | 0.110843 | 0.070231 |
| 37 | 0.07873 | 0.0546 | 0.088904 | 0.067432 | 0.110843 | 0.070231 |
| 38 | 0.07873 | 0.0546 | 0.088904 | 0.067432 | 0.110843 | 0.070231 |
| 39 | 0.07873 | 0.0546 | 0.088904 | 0.067432 | 0.110843 | 0.070231 |
| 40 | 0.010411 | 0.010227 | 0.067393 | 0.025231 | 0.007349 | 0.035829 |
| 41 | 0.010411 | 0.010227 | 0.067393 | 0.025231 | 0.007349 | 0.035829 |
| 42 | 0.010411 | 0.010227 | 0.067393 | 0.025231 | 0.007349 | 0.035829 |
| 43 | 0.010411 | 0.010227 | 0.067393 | 0.025231 | 0.007349 | 0.035829 |
| 44 | 0.010411 | 0.010227 | 0.067393 | 0.025231 | 0.007349 | 0.035829 |
| 45 | 0.010411 | 0.010227 | 0.067393 | 0.025231 | 0.007349 | 0.035829 |
| 46 | 0.010411 | 0.010227 | 0.067393 | 0.025231 | 0.007349 | 0.035829 |
| 47 | 0.010411 | 0.010227 | 0.067393 | 0.025231 | 0.007349 | 0.035829 |
| 48 | 0.010411 | 0.010227 | 0.067393 | 0.025231 | 0.007349 | 0.035829 |
| 49 | 0.010411 | 0.010227 | 0.067393 | 0.025231 | 0.007349 | 0.035829 |
| 50 | 0.010411 | 0.010227 | 0.067393 | 0.025231 | 0.007349 | 0.035829 |
| 51 | 0.010411 | 0.010227 | 0.067393 | 0.025231 | 0.007349 | 0.035829 |
| 52 | 0.010411 | 0.010227 | 0.067393 | 0.025231 | 0.007349 | 0.035829 |
| 53 | 0.010411 | 0.010227 | 0.067393 | 0.025231 | 0.007349 | 0.035829 |
| 54 | 0.010411 | 0.010227 | 0.067393 | 0.025231 | 0.007349 | 0.035829 |
| 55 | 0.010411 | 0.010227 | 0.067393 | 0.025231 | 0.007349 | 0.035829 |
| 56 | 0.011133 | 0.006056 | 0.012375 | 0.01566 | 0.005516 | 0.004206 |
| 57 | 0.011133 | 0.006056 | 0.012375 | 0.01566 | 0.005516 | 0.004206 |
| 58 | 0.011133 | 0.006056 | 0.012375 | 0.01566 | 0.005516 | 0.004206 |
| 59 | 0.011133 | 0.006056 | 0.012375 | 0.01566 | 0.005516 | 0.004206 |
| 60 | 0.011133 | 0.006056 | 0.012375 | 0.01566 | 0.005516 | 0.004206 |
| 61 | 0.011133 | 0.006056 | 0.012375 | 0.01566 | 0.005516 | 0.004206 |
| 62 | 0.011133 | 0.006056 | 0.012375 | 0.01566 | 0.005516 | 0.004206 |
| 63 | 0.011133 | 0.006056 | 0.012375 | 0.01566 | 0.005516 | 0.004206 |
| 64 | 0.011133 | 0.006056 | 0.012375 | 0.01566 | 0.005516 | 0.004206 |
| 65 | 0.011133 | 0.006056 | 0.012375 | 0.01566 | 0.005516 | 0.004206 |
| 66 | 0.011133 | 0.006056 | 0.012375 | 0.01566 | 0.005516 | 0.004206 |

(continued)

Table A-3b. Micro- to Macroalbuminuria Transition Probabilities, African American (Age 30 Macro Prevalence) (continued)

|  | **Neither** | | **Hypertension** | | **Diabetes** | |
| --- | --- | --- | --- | --- | --- | --- |
| **Age** | **Men** | **Women** | **Men** | **Women** | **Men** | **Women** |
| 67 | 0.011133 | 0.006056 | 0.012375 | 0.01566 | 0.005516 | 0.004206 |
| 68 | 0.011133 | 0.006056 | 0.012375 | 0.01566 | 0.005516 | 0.004206 |
| 69 | 0.011133 | 0.006056 | 0.012375 | 0.01566 | 0.005516 | 0.004206 |
| 70 | 0.011133 | 0.006056 | 0.012375 | 0.01566 | 0.005516 | 0.004206 |
| 71 | 0.011133 | 0.006056 | 0.012375 | 0.01566 | 0.005516 | 0.004206 |
| 72 | 0.011133 | 0.006056 | 0.012375 | 0.01566 | 0.005516 | 0.004206 |
| 73 | 0.011133 | 0.006056 | 0.012375 | 0.01566 | 0.005516 | 0.004206 |
| 74 | 0.011133 | 0.006056 | 0.012375 | 0.01566 | 0.005516 | 0.004206 |
| 75 | 0.011133 | 0.006056 | 0.012375 | 0.01566 | 0.005516 | 0.004206 |
| 76 | 0.011133 | 0.006056 | 0.012375 | 0.01566 | 0.005516 | 0.004206 |
| 77 | 0.011133 | 0.006056 | 0.012375 | 0.01566 | 0.005516 | 0.004206 |
| 78 | 0.011133 | 0.006056 | 0.012375 | 0.01566 | 0.005516 | 0.004206 |
| 79 | 0.011133 | 0.006056 | 0.012375 | 0.01566 | 0.005516 | 0.004206 |
| 80 | 0.011133 | 0.006056 | 0.012375 | 0.01566 | 0.005516 | 0.004206 |
| 81 | 0.011133 | 0.006056 | 0.012375 | 0.01566 | 0.005516 | 0.004206 |
| 82 | 0.011133 | 0.006056 | 0.012375 | 0.01566 | 0.005516 | 0.004206 |
| 83 | 0.011133 | 0.006056 | 0.012375 | 0.01566 | 0.005516 | 0.004206 |
| 84 | 0.011133 | 0.006056 | 0.012375 | 0.01566 | 0.005516 | 0.004206 |
| 85 | 0.011133 | 0.006056 | 0.012375 | 0.01566 | 0.005516 | 0.004206 |
| 86 | 0.011133 | 0.006056 | 0.012375 | 0.01566 | 0.005516 | 0.004206 |
| 87 | 0.011133 | 0.006056 | 0.012375 | 0.01566 | 0.005516 | 0.004206 |
| 88 | 0.011133 | 0.006056 | 0.012375 | 0.01566 | 0.005516 | 0.004206 |
| 89 | 0.011133 | 0.006056 | 0.012375 | 0.01566 | 0.005516 | 0.004206 |
| 90 | 0.011133 | 0.006056 | 0.012375 | 0.01566 | 0.005516 | 0.004206 |

Table A-4. Diabetes Incidence (Age 30 Prevalence)

| **Age** | **White Men** | **African American Men** | **Hispanic Men** | **White Women** | **African American Women** | **Hispanic Women** |
| --- | --- | --- | --- | --- | --- | --- |
| 30 | 0.016000 | 0.007000 | 0.002000 | 0.008000 | 0.047000 | 0.026000 |
| 31 | 0.002765 | 0.003599 | 0.003204 | 0.002765 | 0.003599 | 0.003204 |
| 32 | 0.002765 | 0.003599 | 0.003204 | 0.002765 | 0.003599 | 0.003204 |
| 33 | 0.002765 | 0.003599 | 0.003204 | 0.002765 | 0.003599 | 0.003204 |
| 34 | 0.002765 | 0.003599 | 0.003204 | 0.002765 | 0.003599 | 0.003204 |
| 35 | 0.002765 | 0.003599 | 0.003204 | 0.002765 | 0.003599 | 0.003204 |
| 36 | 0.002765 | 0.003599 | 0.003204 | 0.002765 | 0.003599 | 0.003204 |
| 37 | 0.002765 | 0.003599 | 0.003204 | 0.002765 | 0.003599 | 0.003204 |
| 38 | 0.002765 | 0.003599 | 0.003204 | 0.002765 | 0.003599 | 0.003204 |
| 39 | 0.002765 | 0.003599 | 0.003204 | 0.002765 | 0.003599 | 0.003204 |
| 40 | 0.002765 | 0.003599 | 0.003204 | 0.002765 | 0.003599 | 0.003204 |
| 41 | 0.002765 | 0.003599 | 0.003204 | 0.002765 | 0.003599 | 0.003204 |
| 42 | 0.002765 | 0.003599 | 0.003204 | 0.002765 | 0.003599 | 0.003204 |
| 43 | 0.002765 | 0.003599 | 0.003204 | 0.002765 | 0.003599 | 0.003204 |
| 44 | 0.002765 | 0.003599 | 0.003204 | 0.002765 | 0.003599 | 0.003204 |
| 45 | 0.002765 | 0.003599 | 0.003204 | 0.002765 | 0.003599 | 0.003204 |
| 46 | 0.002765 | 0.003599 | 0.003204 | 0.002765 | 0.003599 | 0.003204 |
| 47 | 0.002765 | 0.003599 | 0.003204 | 0.002765 | 0.003599 | 0.003204 |
| 48 | 0.002765 | 0.003599 | 0.003204 | 0.002765 | 0.003599 | 0.003204 |
| 49 | 0.002765 | 0.003599 | 0.003204 | 0.002765 | 0.003599 | 0.003204 |
| 50 | 0.002765 | 0.003599 | 0.003204 | 0.002765 | 0.003599 | 0.003204 |
| 51 | 0.002765 | 0.003599 | 0.003204 | 0.002765 | 0.003599 | 0.003204 |
| 52 | 0.002765 | 0.003599 | 0.003204 | 0.002765 | 0.003599 | 0.003204 |
| 53 | 0.002765 | 0.003599 | 0.003204 | 0.002765 | 0.003599 | 0.003204 |
| 54 | 0.002765 | 0.003599 | 0.003204 | 0.002765 | 0.003599 | 0.003204 |
| 55 | 0.010583 | 0.013774 | 0.012262 | 0.010583 | 0.013774 | 0.012262 |
| 56 | 0.010583 | 0.013774 | 0.012262 | 0.010583 | 0.013774 | 0.012262 |
| 57 | 0.010583 | 0.013774 | 0.012262 | 0.010583 | 0.013774 | 0.012262 |
| 58 | 0.010583 | 0.013774 | 0.012262 | 0.010583 | 0.013774 | 0.012262 |
| 59 | 0.010583 | 0.013774 | 0.012262 | 0.010583 | 0.013774 | 0.012262 |
| 60 | 0.010583 | 0.013774 | 0.012262 | 0.010583 | 0.013774 | 0.012262 |
| 61 | 0.010583 | 0.013774 | 0.012262 | 0.010583 | 0.013774 | 0.012262 |
| 62 | 0.012394 | 0.016132 | 0.014361 | 0.012394 | 0.016132 | 0.014361 |
| 63 | 0.012394 | 0.016132 | 0.014361 | 0.012394 | 0.016132 | 0.014361 |
| 64 | 0.012394 | 0.016132 | 0.014361 | 0.012394 | 0.016132 | 0.014361 |
| 65 | 0.012394 | 0.016132 | 0.014361 | 0.012394 | 0.016132 | 0.014361 |

(continued)

Table A-4. Diabetes Incidence (Age 30 Prevalence) (continued)

| **Age** | **White Men** | **African American Men** | **Hispanic Men** | **White Women** | **African American Women** | **Hispanic Women** |
| --- | --- | --- | --- | --- | --- | --- |
| 66 | 0.012394 | 0.016132 | 0.014361 | 0.012394 | 0.016132 | 0.014361 |
| 67 | 0.012394 | 0.016132 | 0.014361 | 0.012394 | 0.016132 | 0.014361 |
| 68 | 0.012394 | 0.016132 | 0.014361 | 0.012394 | 0.016132 | 0.014361 |
| 69 | 0.012394 | 0.016132 | 0.014361 | 0.012394 | 0.016132 | 0.014361 |
| 70 | 0.012394 | 0.016132 | 0.014361 | 0.012394 | 0.016132 | 0.014361 |
| 71 | 0.012394 | 0.016132 | 0.014361 | 0.012394 | 0.016132 | 0.014361 |
| 72 | 0.012394 | 0.016132 | 0.014361 | 0.012394 | 0.016132 | 0.014361 |
| 73 | 0.012394 | 0.016132 | 0.014361 | 0.012394 | 0.016132 | 0.014361 |
| 74 | 0.012394 | 0.016132 | 0.014361 | 0.012394 | 0.016132 | 0.014361 |
| 75 | 0.012394 | 0.016132 | 0.014361 | 0.012394 | 0.016132 | 0.014361 |
| 76 | 0.012394 | 0.016132 | 0.014361 | 0.012394 | 0.016132 | 0.014361 |
| 77 | 0.012394 | 0.016132 | 0.014361 | 0.012394 | 0.016132 | 0.014361 |
| 78 | 0.012394 | 0.016132 | 0.014361 | 0.012394 | 0.016132 | 0.014361 |
| 79 | 0.012394 | 0.016132 | 0.014361 | 0.012394 | 0.016132 | 0.014361 |
| 80 | 0.012394 | 0.016132 | 0.014361 | 0.012394 | 0.016132 | 0.014361 |
| 81 | 0.012394 | 0.016132 | 0.014361 | 0.012394 | 0.016132 | 0.014361 |
| 82 | 0.012394 | 0.016132 | 0.014361 | 0.012394 | 0.016132 | 0.014361 |
| 83 | 0.012394 | 0.016132 | 0.014361 | 0.012394 | 0.016132 | 0.014361 |
| 84 | 0.012394 | 0.016132 | 0.014361 | 0.012394 | 0.016132 | 0.014361 |
| 85 | 0.012394 | 0.016132 | 0.014361 | 0.012394 | 0.016132 | 0.014361 |
| 86 | 0.012394 | 0.016132 | 0.014361 | 0.012394 | 0.016132 | 0.014361 |
| 87 | 0.012394 | 0.016132 | 0.014361 | 0.012394 | 0.016132 | 0.014361 |
| 88 | 0.012394 | 0.016132 | 0.014361 | 0.012394 | 0.016132 | 0.014361 |
| 89 | 0.012394 | 0.016132 | 0.014361 | 0.012394 | 0.016132 | 0.014361 |
| 90 | 0.012394 | 0.016132 | 0.014361 | 0.012394 | 0.016132 | 0.014361 |
| 91 | 0.012394 | 0.016132 | 0.014361 | 0.012394 | 0.016132 | 0.014361 |
| 92 | 0.012394 | 0.016132 | 0.014361 | 0.012394 | 0.016132 | 0.014361 |
| 93 | 0.012394 | 0.016132 | 0.014361 | 0.012394 | 0.016132 | 0.014361 |
| 94 | 0.012394 | 0.016132 | 0.014361 | 0.012394 | 0.016132 | 0.014361 |
| 95 | 0.012394 | 0.016132 | 0.014361 | 0.012394 | 0.016132 | 0.014361 |
| 96 | 0.012394 | 0.016132 | 0.014361 | 0.012394 | 0.016132 | 0.014361 |
| 97 | 0.012394 | 0.016132 | 0.014361 | 0.012394 | 0.016132 | 0.014361 |
| 98 | 0.012394 | 0.016132 | 0.014361 | 0.012394 | 0.016132 | 0.014361 |
| 99 | 0.012394 | 0.016132 | 0.014361 | 0.012394 | 0.016132 | 0.014361 |
| 100 | 0.012394 | 0.016132 | 0.014361 | 0.012394 | 0.016132 | 0.014361 |

Table A-5. Systolic Blood Pressure and Hypertension

| **CKD Model Two-Part SBP Estimation Equations**  Non African American Men, no CKD SBP constant = 73.588x3 − 90.132x2 + 42.315x + 93.214 SBP slope term = 0.7144x3 − 1.0783x2 + 0.9431x + 0.1719  Non African American Men, CKD SBP constant = 232.11x3 − 335.54x2 + 165.53x + 81.436 SBP slope term = 0.2009x3 − 0.136x2 + 0.285x + 0.3096  Non African American Women, no CKD SBP constant = −25.801x3 + 50.383x2 − 7.1252x + 78.705 SBP slope term = 2.564x3 − 3.6664x2 + 1.8555x + 0.3707  Non African American Women, CKD SBP constant = −63.985x3 + 122.33x2 − 16.475x + 84.82 SBP slope term = 2.0195x3 − 3.1538x2 + 1.6531x + 0.3034  Non African American Men, no CKD SBP constant = 73.588x3 − 90.132x2 + 42.315x + 0.5076 SBP slope term = 0.7144x3 − 1.0783x2 + 0.9431x + 0.1719  Non African American Men, CKD SBP constant = 232.11x3 − 335.54x2 + 165.53x + 0.5076 SBP slope term = 0.2009x3 − 0.136x2 + 0.285x + 0.3096  Non African American Women, no CKD SBP constant = −25.801x3 + 50.383x2 − 7.1252x -1.3767 SBP slope term = 2.564x3 − 3.6664x2 + 1.8555x + 0.3707  Non African American Women, CKD SBP constant = −63.985x3 + 122.33x2 − 16.475x -1.3767 SBP slope term = 2.0195x3 − 3.1538x2 + 1.6531x + 0.3034 |
| --- |

Table A-6. Cholesterol, Total and HDL

| **Total Cholesterol 2-part prediction equations**  Men: Total Cholesterol = -0.0246330922827637age2 +3.03589219416359*age +279.51x3 − 393.36x2 + 267.78x + 50.651  Women: Total Cholesterol = -0.000735126185671987*age3 +0.0998797513603773*age2 -2.87237233098934*age+335.1x3 − 458.67x2 + 284.99x + 128.04  where x = random uniform number between 0 and 1.  **HDL Cholesterol, 1-part prediction equation**  Men: HDL Cholesterol = 80.409x3 − 106.95 x2 + 72.682x + 24.453  Women: HDL Cholesterol = 87.054x3 − 110.27x2 + 77.467x + 30.879  where x = random uniform number between 0 and 1. |
| --- |

Table A-7. Left Ventricular Hypertrophy (LVH)

Initial Prevalence

| **Race/Sex Group** | **Prevalence** |
| --- | --- |
| White Men | 0.16 |
| African American Men | 0.33 |
| Hispanic Men | 0.16 |
| White Women | 0.16 |
| African American Women | 0.33 |
| Hispanic Women | 0.16 |

**LVH One-Time Incidence upon CKD Incidence**

| **Race/Sex Group** | **Incidence** |
| --- | --- |
| White Men | 0.607144048 |
| African American Men | 0.507462687 |
| Hispanic Men | 0.607144048 |
| White Women | 0.607144048 |
| African American Women | 0.507462687 |
| Hispanic Women | 0.607144048 |

Table A-8. ESRD Mortality Rates

|  | **Men** | | | | | | | | |
| --- | --- | --- | --- | --- | --- | --- | --- | --- | --- |
|  | **African American** | | | **White** | | | **Hispanic** | | |
| **Age** | **Diabetes** | **Hyper-tension** | **Other** | **Diabetes** | **Hyper-tension** | **Other** | **Diabetes** | **Hyper-tension** | **Other** |
| 00 to 29 | 101.7 | 21.5 | 32.9 | 55.3 | 41.4 | 16.40369 | 81.4 | 16.8 | 19.5 |
| 30 to 44 | 88.7 | 44 | 76.7 | 86.1 | 43.9 | 30.31999 | 98.4 | 23.6 | 31.6 |
| 45 to 54 | 128.7 | 90.9 | 111.9 | 136.8 | 84.2 | 56.68381 | 111.5 | 50 | 59.7 |
| 55 to 64 | 156.1 | 106.6 | 137.8 | 212 | 153.8 | 104.3994 | 149.9 | 112.2 | 81.9 |
| 65 to 74 | 241 | 181.4 | 213.7 | 323.3 | 266.6 | 204.6787 | 228.6 | 193.3 | 176.8 |
| 75 to 84 | 360.8 | 288.4 | 375.2 | 458 | 395.1 | 353.7896 | 372.9 | 311.8 | 333.9 |
| 85+ | 634.6 | 611.1 | 581.5 | 631.6 | 584 | 587.991 | 587.6 | 554.6 | 450.0 |
|  | **Women** | | | | | | | | |
|  | **African American** | | | **White** | | | **Hispanic** | | |
| **Age** | **Diabetes** | **Hyper-tension** | **Other** | **Diabetes** | **Hyper-tension** | **Other** | **Diabetes** | **Hyper-tension** | **Other** |
| 00 to 29 | 106.8 | 47.8 | 49.3 | 115.1 | 45.5 | 20.9 | 80 | 31.1 | 18.1 |
| 30 to 44 | 103.3 | 63.3 | 75.6 | 83.7 | 51 | 36.6 | 77 | 33.8 | 25.1 |
| 45 to 54 | 148.8 | 94.1 | 104.2 | 140.6 | 92 | 53.8 | 114.7 | 53.6 | 48.6 |
| 55 to 64 | 184.4 | 149.9 | 123.5 | 234.3 | 166.4 | 103.9 | 150 | 101.8 | 81.0 |
| 65 to 74 | 237.9 | 221.7 | 203.3 | 314.9 | 285.2 | 194.5 | 236.6 | 204.7 | 152.8 |
| 75 to 84 | 340.5 | 323.1 | 377.9 | 452.8 | 381.2 | 355.2 | 361.2 | 316.3 | 331.6 |
| 85+ | 504.8 | 483.1 | 632.7 | 574.4 | 546.3 | 521.8 | 491.9 | 466.7 | 462.5 |

Notes: Using RenDER data.

Death rate is per 1,000 patient years.

Assumes African American, white are non-Hispanic.

All modalities, United States only, all diagnoses, 2004 data.

1. Total per capita annual costs = % incident * (ESRD first month cost + 11 * subsequent months) + % nonincident * (12 * subsequent months). [↑](#footnote-ref-2)
